# Identification of Progressive Islet Biomarkers for Type 2 Diabetes by Integrated Transcriptomics and Mendelian Randomization: Alterations Spanning the Normal Glucose Tolerance–Impaired Glucose Tolerance–Type 2 Diabetes Continuum

**DOI:** 10.3390/biomedicines14081850

**Published:** 2026-08-18

**Authors:** Nilupaer Aisikaer, Zaoling Liu, Shuya Cai

**Affiliations:** Department of Epidemiology and Health Statistics, School of Public Health, Xinjiang Medical University, Urumqi 830011, China; nilupar@stu.xjmu.edu.cn (N.A.); m13677507258@163.com (S.C.)

**Keywords:** type 2 diabetes mellitus, impaired glucose tolerance, biomarker panel, mendelian randomization, machine learning, single-cell RNA sequencing, diagnostic model

## Abstract

**Background:** Impaired glucose tolerance (IGT) is the principal prediabetic stage preceding type 2 diabetes mellitus (T2D), yet islet-specific biomarkers capable of tracking progressive molecular changes from normal glucose tolerance (NGT) through IGT to T2D remain unestablished. We sought to identify a multi-gene biomarker panel with monotonically increasing expression and causal support across the full glycemic continuum. **Methods:** Two human islet transcriptomic datasets (GSE76895, GSE164416; *n* = 181: NGT 50, IGT 56, T2D 75) were integrated following ComBat batch correction. Candidate biomarkers were identified at the intersection of limma differential expression and weighted gene co-expression network analysis (WGCNA), then refined through a five-algorithm machine learning consensus (LASSO, random forest, XGBoost, SVM-RFE, elastic net). Progressive expression was assessed by the Jonckheere–Terpstra (J–T) trend test. Two-sample Mendelian randomization (MR) with eQTLGen cis-eQTL instruments, Steiger directionality testing, and Bayesian colocalization provided causal inference. A diagnostic model was internally validated via 1000-iteration bootstrap resampling. Cell-type specificity was verified using single-cell RNA sequencing (GSE200044; 127,919 cells). **Results:** A 12-gene biomarker panel (ALDOB, DKK3, PCOLCE2, KCNE4, INHBA, IRF8, ITGB2, LAPTM5, MYOF, RAMP3, RUNX2, S100A4) was identified, with all members passing Bonferroni-corrected J–T trend testing across the NGT–IGT–T2D axis (*p* ≤ 2.4 × 10^−3^). Notably, a direct IGT-versus-NGT transcriptome-wide comparison (11,948 genes) yielded no significant DEGs after FDR correction, indicating that prediabetic islet signals are subtle and detectable only through progressive trend analysis on preselected candidates. Nevertheless, the mean IGT-stage effect size of the 12 hub genes reached 41.9% of the T2D value, with KCNE4 achieving 94.0% (nominal *p* = 1.75 × 10^−4^), identifying it as the earliest-altered biomarker. Two-sample MR using whole-blood eQTLs suggested protective effects of genetically proxied MYOF (OR 0.999, FDR = 1.68 × 10^−4^) and RUNX2 (OR 0.998, FDR = 1.68 × 10^−4^) on T2D risk, with Steiger testing supporting an expression-to-disease direction (*p* < 10^−36^). However, Bayesian colocalization indicated independent causal variants at both loci (PP.H3 > 0.76, PP.H4 < 0.001), substantially weakening the causal interpretation and suggesting that the MR associations may reflect linkage disequilibrium rather than shared causal biology. The panel achieved a bootstrap-corrected AUC of 0.833 (apparent 0.879) with PR-AUC of 0.951. Single-cell validation confirmed upregulation of 7 hub genes in β cells and revealed cell-type-specific patterns invisible in bulk data, including bidirectional INHBA regulation between β and α cells and progressive α-cell proportion expansion (28.97% → 46.41%). Pathway enrichment converged on three mechanistic axes: extracellular matrix remodeling, immune activation, and autoimmune-like responses, with direct enrichment of the type 1 diabetes pathway (hsa04940). **Conclusions:** This study establishes a 12-gene progressive islet biomarker panel spanning the NGT–IGT–T2D continuum, supported by machine learning robustness, genetic causal evidence, diagnostic modeling, and single-cell biological validation. KCNE4 emerges as a candidate early-warning biomarker for prediabetes, while MYOF and RUNX2 represent causally supported compensatory targets, collectively providing a multilayered foundation for T2D risk stratification and precision intervention. From a clinical perspective, the identification of progressive islet biomarkers at the prediabetic stage provides molecular support for early lifestyle intervention, reinforcing that timely detection and behavioral modification remain the most effective strategies to prevent T2D progression.

## 1. Introduction

Type 2 diabetes mellitus is one of the most prevalent chronic metabolic diseases worldwide. According to the International Diabetes Federation, approximately 589 million adults were living with diabetes in 2024, a figure projected to rise to 853 million by 2050, with T2D accounting for more than 90% of cases [1]. Impaired glucose tolerance represents the principal prediabetic stage of T2D: approximately 70% of individuals with IGT eventually progress to overt disease, and the IGT stage is independently associated with elevated cardiovascular risk and increased all-cause mortality even before frank hyperglycemia develops [2,3]. Lifestyle intervention and pharmacological prevention can reduce the risk of IGT-to-T2D progression by 31–58%. Because T2D is largely driven by modifiable lifestyle factors—including sedentary behavior, caloric excess, and environmental exposures—early identification of at-risk individuals at the prediabetic stage offers a critical window in which behavioral intervention can halt or reverse disease progression [4]. Identifying early molecular alterations at the IGT stage and constructing accurate diagnostic models therefore holds substantial value for the secondary prevention of T2D.

Islet β-cell dysfunction and insulin resistance are the two core pathophysiological drivers of T2D. However, most prior biomarker studies have focused on peripheral blood or whole pancreatic tissue and have lacked islet-specific evidence. The Gene Expression Omnibus has accumulated multiple islet transcriptomic datasets [5], providing a foundation for integrative analysis. WGCNA [6] combined with multi-algorithm machine learning feature selection [7] enables identification of robust hub genes within high-dimensional data, while MR uses genetic variants as instrumental variables to infer causal relationships between exposures and outcomes, circumventing conventional confounding [8,9]. Immune infiltration analysis further helps elucidate the contribution of low-grade islet inflammation to T2D progression [10,11].

The mechanistic overlap between T2D and type 1 diabetes mellitus (T1D) at the islet immune level has attracted growing interest. Eizirik et al. [12] proposed that T2D islets harbor a low-grade autoimmune component, and Donath et al. [10] demonstrated that chronic inflammation drives β-cell functional loss through mediators including interleukin-1β and tumor necrosis factor-α. Nevertheless, most prior studies have been limited to bulk transcriptomics without cell-type resolution, and few have integrated multi-omics approaches with causal inference within a single analytical framework.

## 2. Methods

### 2.1. Data Sources

Islet transcriptomic datasets were screened from the Gene Expression Omnibus. GSE76895 (platform GPL570, Affymetrix Human Genome U133 Plus 2.0 Array: Affymetrix, Santa Clara, CA, USA) and GSE164416 (platform GPL16791Illumina HiSeq 4000: Illumina, San Diego, CA, USA) were included as the training set for integrated analysis [13,14]. Remaining candidate datasets were excluded because of insufficient sample size, tissue-type mismatch, or incomplete phenotypic annotation. The single-cell validation set was GSE200044, a human islet scRNA-seq dataset comprising 127,919 cells across three groups (NGT, prediabetic T2D [PreT2D], and T2D) [15]. All data were obtained from publicly available repositories, and ethical approval was therefore not required.

### 2.2. Sample Selection and Grouping

Samples were grouped according to the 2022 American Diabetes Association diagnostic criteria [16], based on 2 h plasma glucose measured during the oral glucose tolerance test (OGTT) and fasting plasma glucose: NGT, fasting glucose < 6.1 mmol/L and 2 h glucose < 7.8 mmol/L; IGT, 2 h glucose 7.8–11.0 mmol/L; T2D, fasting glucose ≥ 7.0 mmol/L or 2 h glucose ≥ 11.1 mmol/L. The original GSE164416 dataset contained 3 samples with impaired fasting glucose (IFG); because the IFG threshold overlaps with that of IGT and the sample size was insufficient to form an independent group, these were excluded during quality control. The final training set comprised 181 samples: NGT 50 (GSE76895, 32; GSE164416, 18), IGT 56 (GSE76895, 15; GSE164416, 41), and T2D 75 (GSE76895, 36; GSE164416, 39).

In the primary analysis, IGT and T2D were combined into a disease group (*n* = 131) and compared with the NGT control group (*n* = 50) to increase statistical power. Progressive alterations were additionally evaluated through an independent IGT (*n* = 56) versus NGT (*n* = 50) differential analysis and a three-group J–T trend test (NGT/IGT/T2D).

### 2.3. Data Preprocessing and Batch Correction

**Figure 1:**
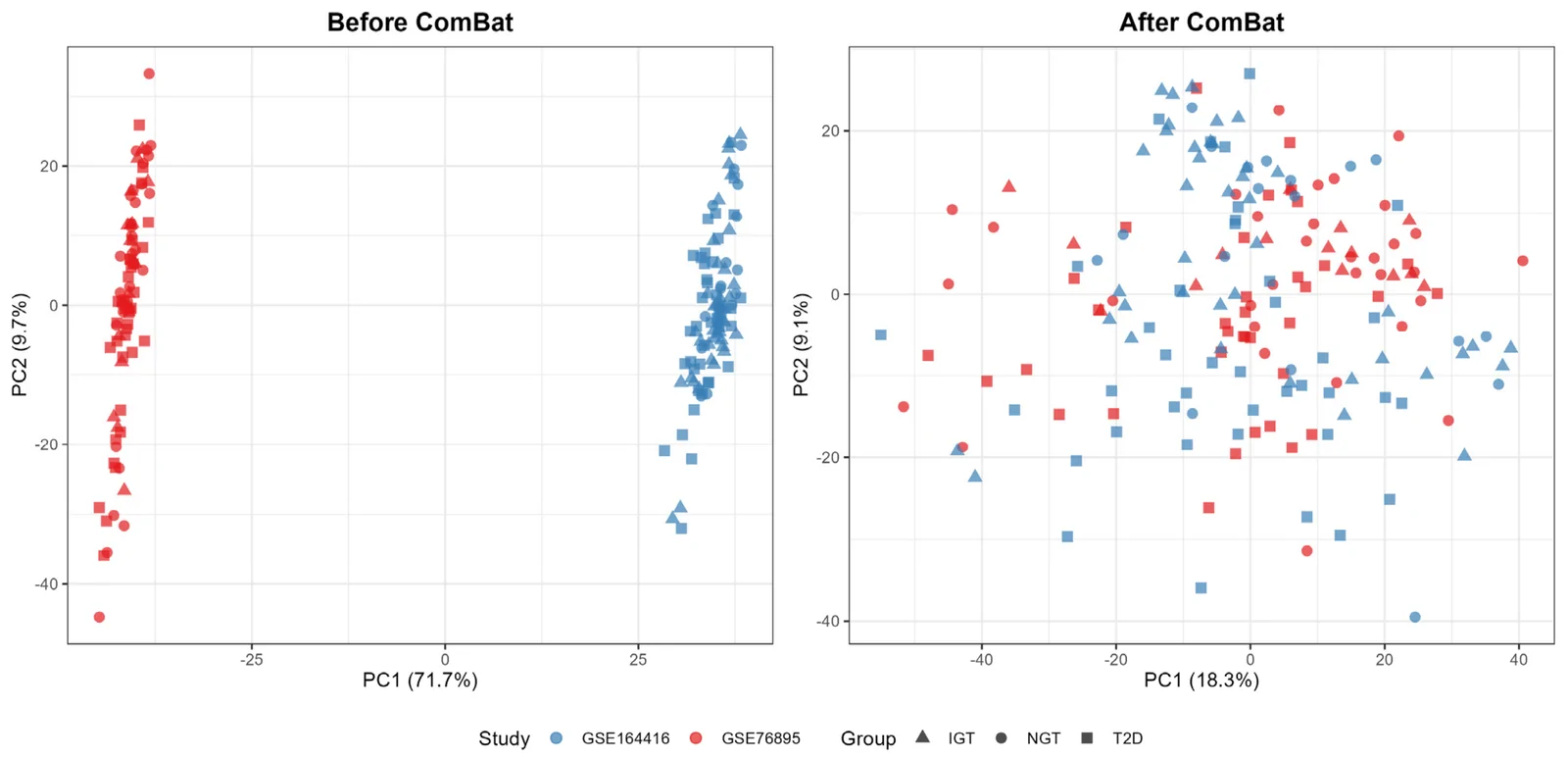
Principal component analysis before and after ComBat batch correction. (**Left**), before correction; samples cluster by dataset origin. (**Right**), after correction; samples mix while disease grouping is preserved. Axes show the first two principal components with variance explained in parentheses.

Raw CEL files from GSE76895 were normalized using the robust multi-array average (RMA) algorithm, and RNA-seq counts from GSE164416 were transformed using voom. After intersecting the two datasets by ENSEMBL ID, 11,948 shared genes were retained. Batch effects were removed using the ComBat function of the sva package [17], and correction efficacy was assessed by principal component analysis (PCA; Figure 1).

Before correction, samples cluster distinctly by dataset origin (GSE76895 vs. GSE164416), indicating substantial batch effects. After ComBat correction, samples from the two datasets are well intermixed, whereas the three disease groups (NGT, IGT, T2D) remain discernible, confirming that biological variation was preserved while technical variation was removed. Each point represents one sample, colored by dataset or disease group. Numbers in parentheses indicate the percentage of variance explained by each principal component. NGT, normal glucose tolerance; IGT, impaired glucose tolerance; T2D, type 2 diabetes mellitus.

### 2.4. Differential Expression Analysis

Differential expression analysis was performed using the limma package [18] under two frameworks: (i) a primary analysis comparing the disease group (IGT + T2D, *n* = 131) versus NGT (*n* = 50); and (ii) an IGT subgroup analysis comparing IGT (*n* = 56) versus NGT (*n* = 50), with dataset origin included as a covariate to control for residual batch effects. Differentially expressed genes (DEGs) were defined by |log_2_FC| > 0.5 and FDR < 0.05.

### 2.5. Weighted Gene Co-Expression Network Analysis

A scale-free co-expression network was constructed using the top 50% most variable genes (5000 genes). The soft-thresholding power was determined by the pickSoftThreshold function (β = 10, R^2^ > 0.85). Modules were identified by dynamic tree cutting (minimum module size, 30; module eigengene similarity merging threshold, 0.25). Pearson correlations between module eigengenes and the NGT/IGT/T2D phenotypes were calculated, and modules with *p* < 0.05 were designated as core modules. Genes with module membership > 0.7 and gene significance > 0.4 were defined as module hub genes; their intersection with DEGs constituted the candidate gene set.

### 2.6. Machine Learning Identification of Hub Genes

The 74 candidate genes (DEG ∩ WGCNA) were subjected to hub-gene voting across five algorithms: least absolute shrinkage and selection operator (LASSO) regression (glmnet; λ.min selected by 10-fold cross-validation) [19]; random forest (randomForest; ntree = 500, ranked by MeanDecreaseGini) [20]; XGBoost (xgboost; nrounds = 200, eta = 0.1, ranked by Gain) [21] cursive feature elimination (SVM-RFE; caret, 5-fold cross-validation) [22]; and elastic net (glmnet, α = 0.5). Each algorithm retained its top 30 genes, and the 12 genes with the highest voting frequency constituted the hub-gene set.

To assess robustness, the top-N cutoff for random forest, XGBoost, and SVM-RFE (N = 5–50, step 5) and the voting threshold (selected by ≥K algorithms; K = 2–5) were varied, generating 40 parameter combinations. LASSO (3 features) and elastic net (4 features) outputs were fixed by their intrinsic criteria. For each combination, agreement with the original 12 hub genes was quantified by the Jaccard similarity index, and per-gene selection frequency across the 40 combinations was computed (Table S1).

### 2.7. Jonckheere–Terpstra Trend Test

To evaluate progressive alterations along the NGT → IGT → T2D axis, the J–T monotonic-increasing trend test (DescTools; 10,000 permutations) was applied to each hub gene across the three groups. Multiple comparisons were corrected by both the Benjamini–Hochberg (BH) and Bonferroni methods. The minimum resolvable *p*-value of the permutation test is 1/10,000 = 10^−4^; *p*_trend < 10^−4^ indicates that no null permutation exceeded the observed value. To contextualize the hub-gene results, the same J–T test was applied to all 11,948 expressed genes; the resulting *p*-value distribution was used to assess the specificity of the hub-gene monotonic trends (Supplementary File: Figures S2 and S3).

### 2.8. Mendelian Randomization Analysis

Two-sample MR was performed to assess the causal effect of hub-gene expression on T2D. Exposure instruments were drawn from eQTLGen whole-blood cis-eQTL data [9], and the outcome was the Suzuki et al. 2024 T2D GWAS (European ancestry) [23]. Instruments were required to: (i) be associated with the exposure (cis-eQTL *p* < 5 × 10^−8^); (ii) lie within ±1 Mb of the transcription start site; (iii) be mutually independent (linkage disequilibrium [LD] r^2^ < 0.001, distance > 10,000 kb); (iv) have a corresponding effect estimate in the outcome GWAS; and (v) pass harmonization with palindromic single-nucleotide polymorphisms (SNPs) removed. Because *p* < 5 × 10^−8^ corresponds to |Z| > 5.45, the F statistic (F = Z^2^) of all instruments exceeded 29.7, well above the threshold for a strong instrument; weak-instrument bias was therefore negligible.

Of the 12 hub genes, KCNE4, INHBA, IRF8, and S100A4 lacked genome-wide significant cis-eQTL instruments and were excluded; the remaining 8 (MYOF, RUNX2, RAMP3, DKK3, ITGB2, LAPTM5, PCOLCE2, ALDOB) entered MR. Genes with ≥2 instruments were analyzed by inverse-variance weighting (IVW) [24], and single-instrument genes by the Wald ratio. With fewer than 3 instruments, MR-Egger intercept and Cochran’s Q tests were not feasible; horizontal pleiotropy sensitivity analyses were therefore not performed for single-instrument genes. MR *p*-values across the 8 genes were BH-corrected, with FDR < 0.05 considered significant. MR was performed using the TwoSampleMR package v0.5.7 [25]; Steiger testing was implemented in R; colocalization used the coloc package v5.2.3 [26,27]. Instrument details for MYOF and RUNX2 are provided in Supplementary File: Table S2.

For the MR-significant genes (MYOF, RUNX2), the Steiger test computed per-instrument variance explained for the exposure (R^2^_exposure = Z^2^/[Z^2^ + N]) and outcome (R^2^_outcome = χ^2^/N_eff); R^2^_exposure significantly exceeding R^2^_outcome (Steiger Z test, *p* < 0.05) supported a gene-expression-to-T2D causal direction [28]. Bayesian colocalization evaluated whether eQTL and GWAS signals shared a causal variant. Full cis-eQTL summary statistics were obtained from eQTLGen (released 2019; N ≤ 31,684; whole blood) [9]. All SNPs within the cis window were included using default priors (p1 = p2 = 1 × 10^−4^, p12 = 1 × 10^−5^). PP.H4 ≥ 0.75 indicated a shared causal variant; PP.H3 > 0.5 indicated independent causal variants. Sensitivity of colocalization conclusions to the p12 prior was evaluated across a range of 1 × 10^−6^ to 1 × 10^−4^ (Supplementary File: Table S3).

### 2.9. Diagnostic Model Construction and Internal Validation

The 12 hub genes were entered into a multivariable logistic regression model (disease group vs. NGT), and a nomogram was generated (rms package) [29]. Performance was assessed by the receiver operating characteristic (ROC) curve, area under the curve (AUC), precision-recall AUC (PR-AUC), Brier score, calibration curve, and decision curve analysis (DCA) [30,31,32]. Because the training-set events per variable (EPV = 50/12 = 4.17) was below the recommended threshold of 10, internal validation used 1000 bootstrap resamples via the validate() function to estimate optimism and report optimism-corrected Somers’ Dxy with the corresponding AUC (AUC = [Dxy + 1]/2), calibration slope, and Brier score. The calibration curve used 200 bootstrap resamples. Given class imbalance (disease:NGT ≈ 2.6:1), PR-AUC was reported as a supplementary metric, with a random baseline of 0.724.

### 2.10. Immune Infiltration Analysis

The single-sample gene set enrichment analysis (ssGSEA) algorithm of the GSVA package [33] quantified immune-infiltration scores for 24 immune cell types using the marker sets of Charoentong et al. [34]. Differences between the disease and NGT groups were compared (Wilcoxon test, FDR < 0.05), and Spearman correlations between hub-gene expression and immune scores were calculated.

### 2.11. Pathway Enrichment Analysis

Gene Ontology (GO; biological process [BP], cellular component, molecular function) and Kyoto Encyclopedia of Genes and Genomes (KEGG) enrichment were performed separately for the 74 candidate genes and the 12 hub genes. SYMBOL-to-ENTREZID conversion used org.Hs.eg.db v3.18; all genes converted successfully. GO enrichment used clusterProfiler::enrichGO(). KEGG enrichment used a local TERM2GENE mapping from the org.Hs.eg.db PATH table (229 hsa pathways, 5868 genes) with hypergeometric testing via clusterProfiler::enricher() [35], BH-corrected. Candidate-gene thresholds were *p* < 0.05 and q < 0.2; hub-gene thresholds were *p* < 0.1 and q < 0.25. Results were visualized with enrichplot v1.20.

### 2.12. Single-Cell Validation

GSE200044 was processed with the Seurat 4.3 workflow [36]: quality control (200–6000 detected genes per cell; mitochondrial proportion < 20%), SCTransform normalization, Harmony integration [37], and uniform manifold approximation and projection (UMAP; first 30 principal components, resolution 0.5). Ten cell types were annotated by canonical islet markers (INS for β, GCG for α, SST for δ, PPY for γ, KRT19 for ductal, CPA1 for acinar, PECAM1 for endothelial, PDGFRA for stellate, PTPRC for immune cells). After integration, 127,919 cells were obtained (NGT 31,884; PreT2D 46,704; T2D 49,331), including 43,538 β cells and 53,007 α cells. Differential expression of the 12 hub genes across the three groups was analyzed separately in β and α cells (Seurat::FindMarkers, Wilcoxon test), BH-corrected (38,107 genes per comparison). Cell-type composition differences were assessed by the Kruskal–Wallis test with Dunn’s post hoc test.

### 2.13. Statistical Analysis

All analyses were conducted in R 4.3.1 (R Foundation for Statistical Computing, Vienna, Austria). Unless otherwise stated, group comparisons used the Wilcoxon rank-sum test, and multiple comparisons were corrected by the BH method. Statistical significance was set at two-tailed *p* < 0.05.

## 3. Results

### 3.1. Batch Correction and Data Integration

After integrating GSE76895 and GSE164416, 181 samples and 11,948 shared genes were obtained. Before ComBat correction, PCA showed clear clustering by dataset origin; after correction, samples from the two datasets were well mixed while disease structure was preserved (Figure 1). The final training set comprised 50 NGT, 56 IGT, and 75 T2D samples (Table 1).

Available donor characteristics from GSE76895 (*n* = 83) are summarized in Table 1B. Age, BMI, and sex did not differ significantly among the three glucose tolerance groups (all *p* > 0.19), whereas fasting glucose was significantly elevated in the T2D group (8.0 ± 2.7 vs. 5.3 ± 0.6 mmol/L; *p* = 1.9 × 10^−7^), consistent with the clinical classification. GSE164416 did not deposit individual-level clinical metadata in GEO. The glucose tolerance group distribution differed between datasets (χ^2^ *p* = 6 × 10^−4^; GSE164416 contributed proportionally more IGT samples); dataset origin was included as a covariate in the differential expression model and ComBat batch correction to mitigate potential confounding.

### 3.2. Differential Expression Analysis

**Figure 2:**
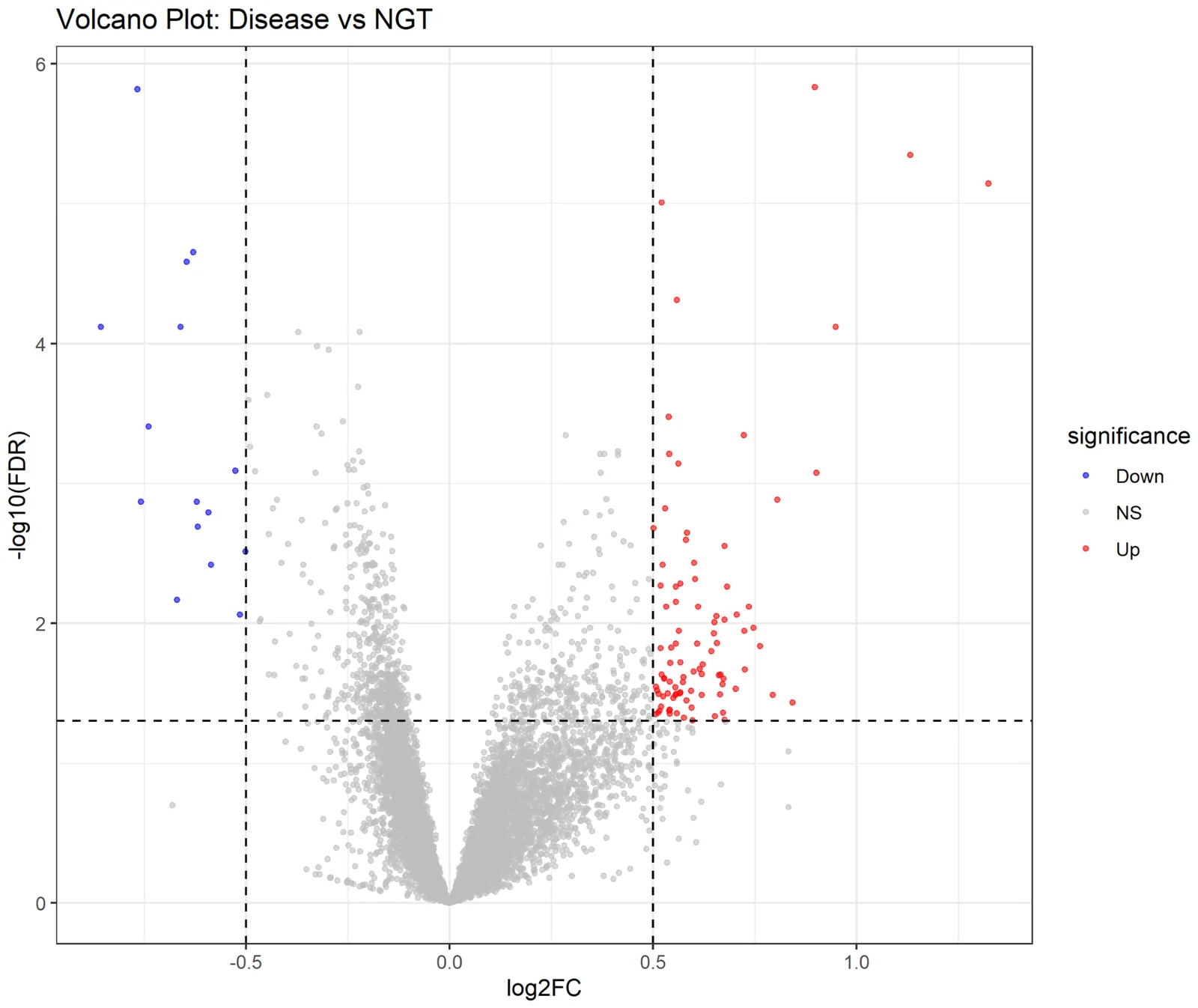
Volcano plot of differentially expressed genes (disease group vs. NGT meta-analysis). The x-axis shows log_2_ fold change and the y-axis shows −log_10_ FDR. Red dots represent significantly upregulated genes (*n* = 92; log_2_FC > 0.5, FDR < 0.05); blue dots represent significantly downregulated genes (*n* = 15; log_2_FC < −0.5, FDR < 0.05); grey dots represent non-significant genes. Dashed lines indicate the applied thresholds. The 12 hub genes identified by subsequent machine learning analysis are labeled. Dataset origin was included as a covariate. NGT, normal glucose tolerance; FDR, false discovery rate.

The disease group (*n* = 131) versus NGT (*n* = 50) comparison identified 107 DEGs (92 upregulated and 15 downregulated; Figure 2). The upregulated genes were functionally enriched for three themes: extracellular matrix remodeling (e.g., PCOLCE2, log_2_FC = +1.13; DKK3, +0.90; ALDOB, +1.32), immune/inflammatory signaling (e.g., IRF8, +0.72; ITGB2, +0.60; LAPTM5, +0.68), and developmental regulation (e.g., RUNX2, +0.58). The 15 downregulated DEGs included canonical β-cell functional markers such as SLC2A2 (log_2_FC = −1.46) and ARG2 (−0.77), consistent with loss of mature β-cell identity in T2D.

### 3.3. WGCNA Co-Expression Network

**Figure 3:**
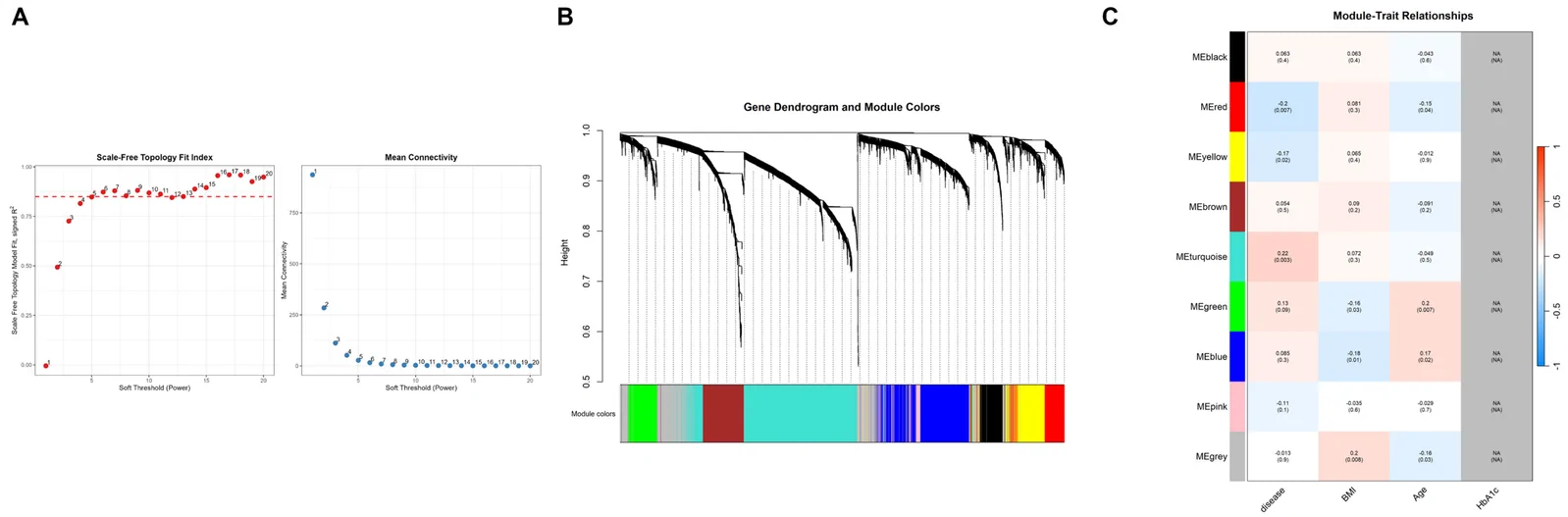
Weighted gene co-expression network analysis (WGCNA). (**A**) Soft-thresholding power selection. The scale-free topology fit index (R^2^) and mean connectivity are plotted against candidate powers; β = 10 was selected (R^2^ > 0.85). (**B**) Hierarchical clustering dendrogram of co-expressed gene modules with merged color assignments; nine modules were identified. (**C**) Module eigengene–trait correlation heatmap. Pearson correlation coefficients and corresponding *p*-values are shown for each module–trait pair. The turquoise module (1462 genes) was positively correlated with disease status (r = 0.22, *p* = 3.4 × 10^−3^), and the red module (281 genes) was negatively correlated (r = −0.20, *p* = 6.8 × 10^−3^). NGT, normal glucose tolerance; IGT, impaired glucose tolerance; T2D, type 2 diabetes mellitus.

Nine modules were identified (Figure 3B). Three modules were significantly correlated with disease status (*p* < 0.05): turquoise (1462 genes; r = 0.22, *p* = 3.4 × 10^−3^), positively correlated with T2D progression; red (281 genes; r = −0.20, *p* = 6.8 × 10^−3^), negatively correlated; and yellow (414 genes; r = −0.17, *p* = 0.025). The turquoise and red modules, representing the most significant disease-associated modules with opposing directions, were selected as core modules, together yielding 1743 core-module genes (Figure 3C).

### 3.4. Candidate Gene Identification

**Figure 4:**
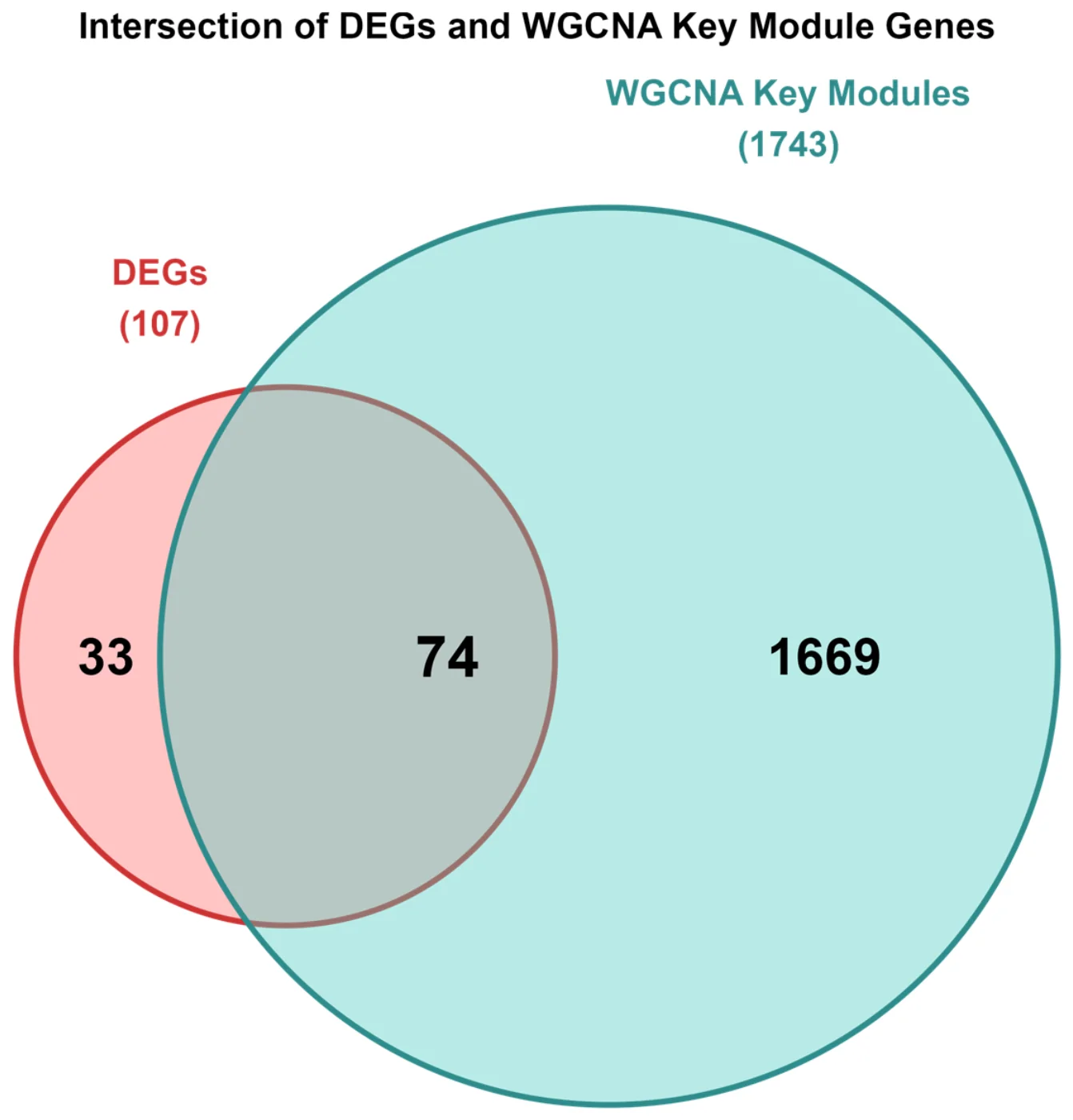
Venn diagram of candidate gene identification. Overlap between differentially expressed genes (DEGs, *n* = 107; disease group vs. NGT) and WGCNA core-module genes (*n* = 1743; turquoise + red modules). The intersection yielded 74 candidate genes, all belonging to the turquoise module, that were carried forward into machine learning feature selection. DEG, differentially expressed gene; WGCNA, weighted gene co-expression network analysis; NGT, normal glucose tolerance.

The intersection of DEGs and WGCNA core-module genes yielded 74 candidate genes (Figure 4). All 74 candidates belonged to the turquoise module and were upregulated in the disease group (log_2_FC range: +0.50 to +1.32). No red-module genes met the dual DEG threshold (FDR < 0.05 and |log_2_FC| > 0.5), attributable to the small module size (281 genes) and the predominance of upregulated DEGs (92 of 107). This directional homogeneity reflects the co-expression architecture: the turquoise module’s positive correlation with disease status inherently enriches for genes upregulated in T2D islets.

### 3.5. Machine Learning Identification of Hub Genes

**Figure 5:**
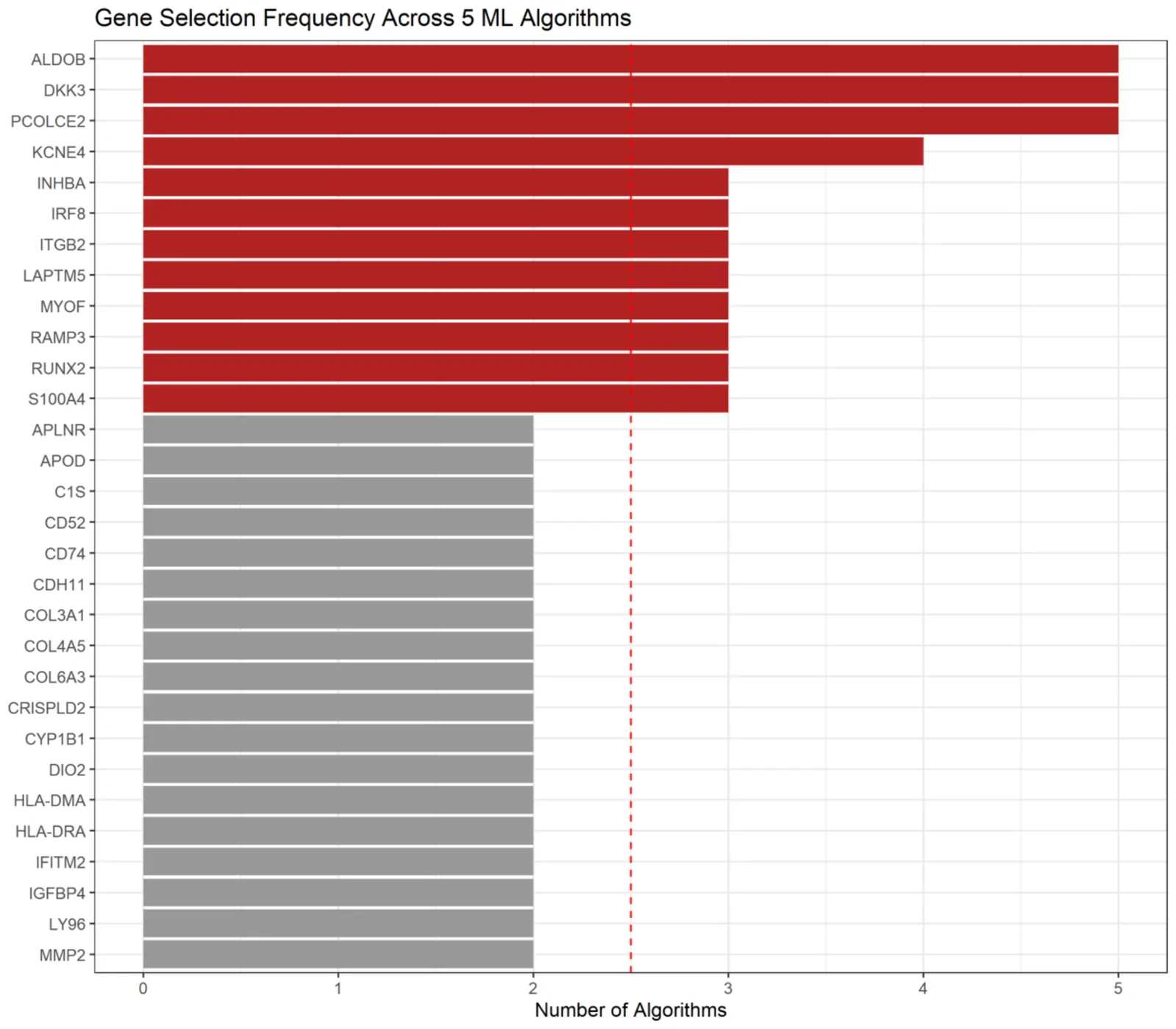
Hub-gene identification by multi-algorithm machine learning voting. Bar chart showing the selection frequency of each candidate gene across five feature-selection algorithms: LASSO regression, random forest (ranked by MeanDecreaseGini), XGBoost (ranked by Gain), SVM-RFE, and elastic net. Each algorithm nominated its top 30 genes; genes selected by ≥3 of the 5 algorithms (under the primary parameter setting, top-50/vote ≥ 3) constituted the 12-gene hub-gene panel (highlighted). The vertical red dashed line represents the voting threshold (visually set at 2.5) to distinguish the robust hub genes (selected by ≥3 algorithms, shown as red bars) from those with lower consistency (selected by only 2 algorithms, shown as grey bars). LASSO, least absolute shrinkage and selection operator; SVM-RFE, support vector machine recursive feature elimination. Hub-gene voting frequency across five machine learning algorithms. Feature-selection frequencies of LASSO, random forest, XGBoost, SVM-RFE, and elastic net; the top 12 constitute the hub-gene panel.

After ranking the 74 candidate genes across the five algorithms and intersecting the results (Figure 5), 12 hub genes were identified: ALDOB, DKK3, PCOLCE2, KCNE4, INHBA, IRF8, ITGB2, LAPTM5, MYOF, RAMP3, RUNX2, and S100A4 (Table 2), all upregulated in T2D versus NGT (FDR < 0.05).

Sensitivity analysis across the 40 parameter combinations showed that, under the primary parameters (top-50, vote ≥ 3), the 12 hub genes were selected identically (Jaccard = 1.0; Table 3 and Supplementary File: Table S1). For top-N ≥ 20 and vote ≥ 3, the Jaccard index exceeded 0.75 throughout. ALDOB, DKK3, and PCOLCE2 were selected in 100% of combinations, and KCNE4 in 75%. MYOF (37.5%) and RUNX2 (42.5%) showed lower stability, reflecting intermediate within-algorithm ranking; however, their causal roles were independently validated by MR (FDR = 1.68 × 10^−4^) and Steiger testing (*p* < 10^−36^), independent of machine learning thresholds.

Top-N for RF, XGBoost, and SVM-RFE (N = 5–50, step 5) and the voting threshold (≥K algorithms, K = 2–5) were varied, giving 40 combinations; LASSO (3 features) and elastic net (4 features) were fixed. Highly stable, selected in ≥80%; moderately stable, 50–80%; threshold-sensitive, <50%. Under the primary parameters, Jaccard = 1.0; for top-N ≥ 20 and vote ≥ 3, Jaccard > 0.75 throughout.

### 3.6. Progressive Alterations of Hub Genes Along the Diabetes Progression Axis

To determine whether the hub-gene alterations represent terminal T2D events or continuous changes along the progression axis, we examined two complementary perspectives.

#### 3.6.1. Igt Versus Ngt Subgroup Analysis

**Figure 6:**
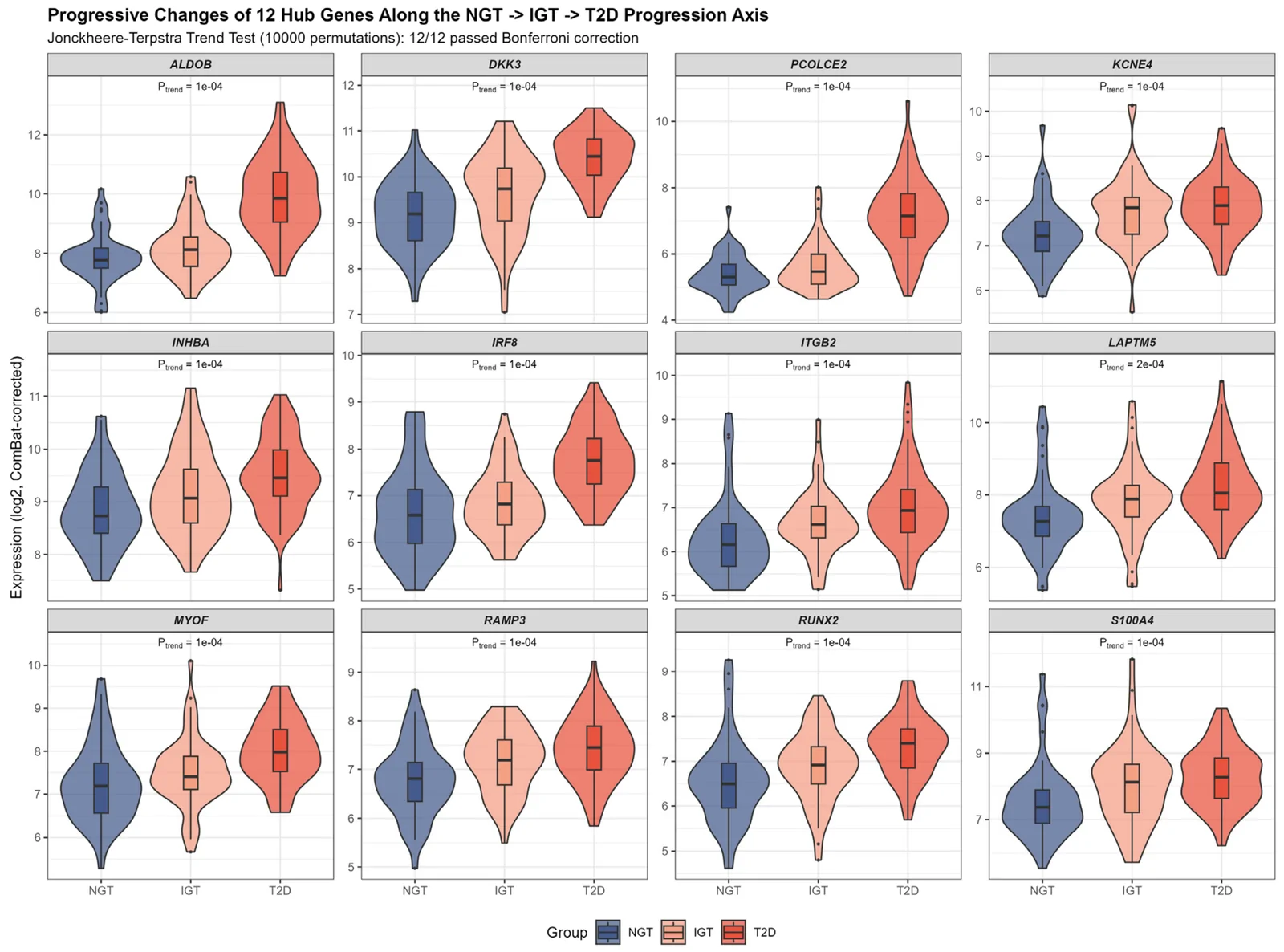
Progressive expression of the 12 hub genes along the NGT → IGT → T2D continuum. Violin plots display the distribution of normalized expression for each hub gene across the three groups (NGT, IGT, T2D; *n* = 50, 56, 75, respectively). The inner box plot shows the median and interquartile range. Jonckheere–Terpstra (J–T) trend test *p*-values (10,000 permutations) are annotated; all 12 genes passed Bonferroni correction (*p*_Bonf ≤ 2.4 × 10^−3^), confirming a monotonically increasing trend across the glycemic continuum. NGT, normal glucose tolerance; IGT, impaired glucose tolerance; T2D, type 2 diabetes mellitus. Violin plots of the 12 hub genes along the NGT → IGT → T2D axis. Each panel shows one gene; groups are color-coded. Labels indicate J–T trend test *p*-values. All 12 passed Bonferroni correction.

Differential analysis of IGT (*n* = 56) versus NGT (*n* = 50) used limma with Study as a covariate (|log_2_FC| > 0.5, FDR < 0.05). In the transcriptome-wide comparison (11,948 genes), no DEG reached this threshold, indicating that IGT-stage transcriptomic changes are milder than at T2D (Supplementary File: Figure S1). However, focusing on the 12 hub genes revealed a clear progressive pattern (Table 4, Figure 6).

All 12 genes shared the same direction (upregulated) at IGT and T2D, with no reversal. The IGT-stage log_2_FC ranged from 0.172 to 0.580 (mean 0.379), all smaller than the corresponding T2D values (0.617–2.053, mean 1.061). The mean IGT effect size reached 41.9% of the T2D value, a typical dose-gradient pattern. Although no gene reached significance after FDR correction, 7 hub genes reached nominal *p* < 0.05 at IGT (DKK3, KCNE4, INHBA, ITGB2, LAPTM5, RAMP3, S100A4). Notably, KCNE4 reached 94.0% of its T2D effect size at IGT (log_2_FC 0.580 vs. 0.617; *p* = 1.75 × 10^−4^), and S100A4 (66.8%), RAMP3 (64.4%), and INHBA (60.1%) exceeded 50%.

#### 3.6.2. Three-Group Jonckheere–Terpstra Trend Test

The J–T monotonic-increasing trend test (10,000 permutations) across the NGT, IGT, and T2D groups showed that all 12 hub genes increased monotonically along the progression axis (Table 5): 11 genes had *p*_trend < 10^−4^ (the permutation precision limit) and LAPTM5 had *p*_trend ≤ 2 × 10^−4^. After BH-FDR correction, all 12 remained significant (FDR ≤ 2.0 × 10^−4^), as did all 12 after Bonferroni correction (*p*_Bonf ≤ 2.4 × 10^−3^). To contextualize these results, the same J–T test (increasing alternative, 10,000 permutations) was applied transcriptome-wide to all 11,948 expressed genes. Of these, 1365 (11.4%) reached the Bonferroni threshold of *p* ≤ 0.05/12, and 428 (3.6%) attained the permutation floor of *p* ≤ 1 × 10^−4^ (Supplementary File: Figures S2 and S3). All 12 hub genes achieved *p* ≤ 1 × 10^−4^, ranking within the top 0.13–2.69% of the transcriptome. Thus, although monotonic increasing trends are not unique to the hub genes, the 12 selected biomarkers reside in the extreme tail of the genome-wide distribution, supporting—but not independently confirming—their progressive behavior along the NGT–IGT–T2D axis. Taken together, the two analyses indicate that the hub genes are not only T2D diagnostic markers but also continuous molecular events throughout progression. KCNE4 reached 94% of its T2D effect size at IGT, suggesting distinctive potential for early identification of prediabetes.

J–T test based on 10,000 permutations under the alternative hypothesis of monotonic increase (NGT < IGT < T2D); *p*_trend < 10^−4^ indicates the permutation precision limit. FDR correction used BH (*n* = 12); Bonferroni based on 12 tests. All 12 hub genes passed FDR < 0.05 and Bonferroni < 0.05.

### 3.7. Mendelian Randomization Causal Validation

**Figure 7:**
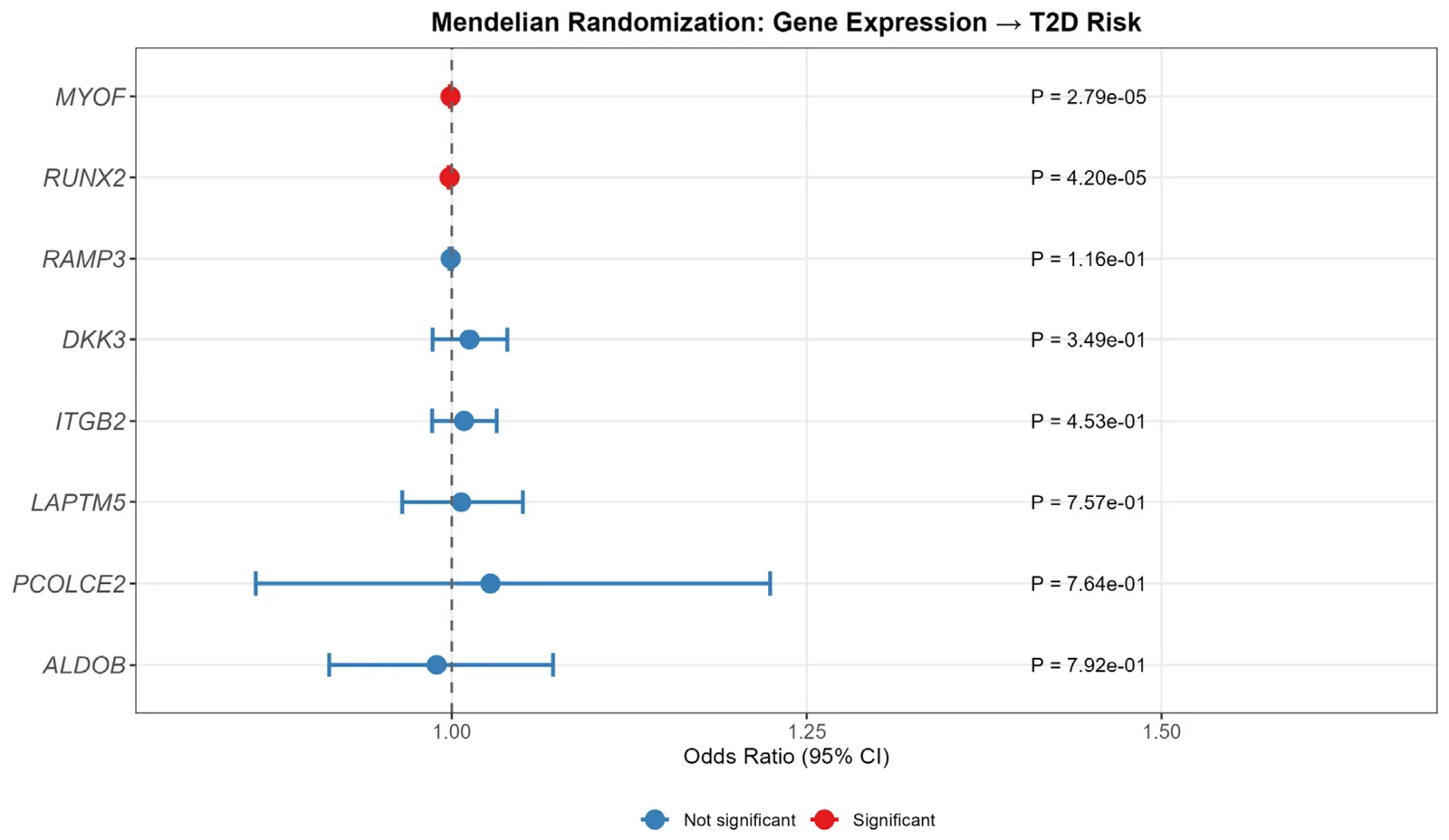
Forest plot of Mendelian randomization causal effect estimates for eight hub genes on T2D risk. Forest plot of Mendelian randomization causal effects for hub genes. X-axis, odds ratio (95% CI); red dashed line, odds ratio = 1. MYOF and RUNX2 remained significant after BH-FDR correction, indicating protective effects. All instruments had F > 29.7.

MR was performed for the 12 hub genes. Of these, 8 had genome-wide significant cis-eQTLs as instruments; 4 (KCNE4, INHBA, IRF8, S100A4) were excluded for lack of instruments. After LD clumping, the 8 genes yielded 11 independent instruments. MYOF (2 instruments) had an IVW odds ratio of 0.999 (95% CI 0.999–1.000; *p* = 2.79 × 10^−5^), suggesting a statistically significant inverse association between genetically proxied MYOF expression and T2D risk. RUNX2 (2 instruments) had an IVW odds ratio of 0.998 (95% CI 0.998–0.999; *p* = 4.20 × 10^−5^), likewise showing an inverse association. The remaining 6 genes were non-significant (*p* > 0.05; Table 6, Figure 7).

For the FDR-significant genes, the Steiger test confirmed a gene-expression-to-T2D direction (Table 7): for both MYOF instruments, R^2^_exposure greatly exceeded R^2^_outcome (combined ratio 1855; Steiger Z = 23.49; *p* = 5.41 × 10^−122^), and RUNX2 likewise (combined ratio 4612; Steiger Z = 41.75; *p* < 10^−300^). All 4 instruments were directionally consistent and significant (*p* < 7 × 10^−4^), excluding reverse causality. Colocalization indicated independent causal variants at both loci (MYOF: 6306 SNPs, PP.H3 ≈ 1.00, PP.H4 ≈ 0; RUNX2: 6275 SNPs, PP.H3 = 0.76, PP.H4 = 0.0006). The p12 sensitivity analysis (1 × 10^−6^ to 1 × 10^−4^) showed stable conclusions (Supplementary File: Table S3). These findings suggest the MR effect may be influenced by LD and should be interpreted cautiously given that eQTLGen reflects whole blood rather than islet tissue (see Discussion).

Each row represents one gene; the point estimate (odds ratio per one-unit increase in genetically predicted gene expression) and 95% confidence interval are shown. The red dashed vertical line marks OR = 1 (null). Genes with ≥2 instruments were analyzed by inverse-variance weighting (IVW); single-instrument genes by the Wald ratio. MYOF and RUNX2 remained significant after Benjamini–Hochberg FDR correction (FDR = 1.68 × 10^−4^), indicating protective causal effects. All instruments had an F-statistic > 29.7. Exposure: eQTLGen whole-blood cis-eQTLs; outcome: Suzuki et al. 2024 T2D GWAS (European ancestry) [23]. T2D, type 2 diabetes mellitus; IVW, inverse-variance weighted; OR, odds ratio; CI, confidence interval; FDR, false discovery rate.

### 3.8. Diagnostic Model Construction and Internal Validation

**Figure 8:**
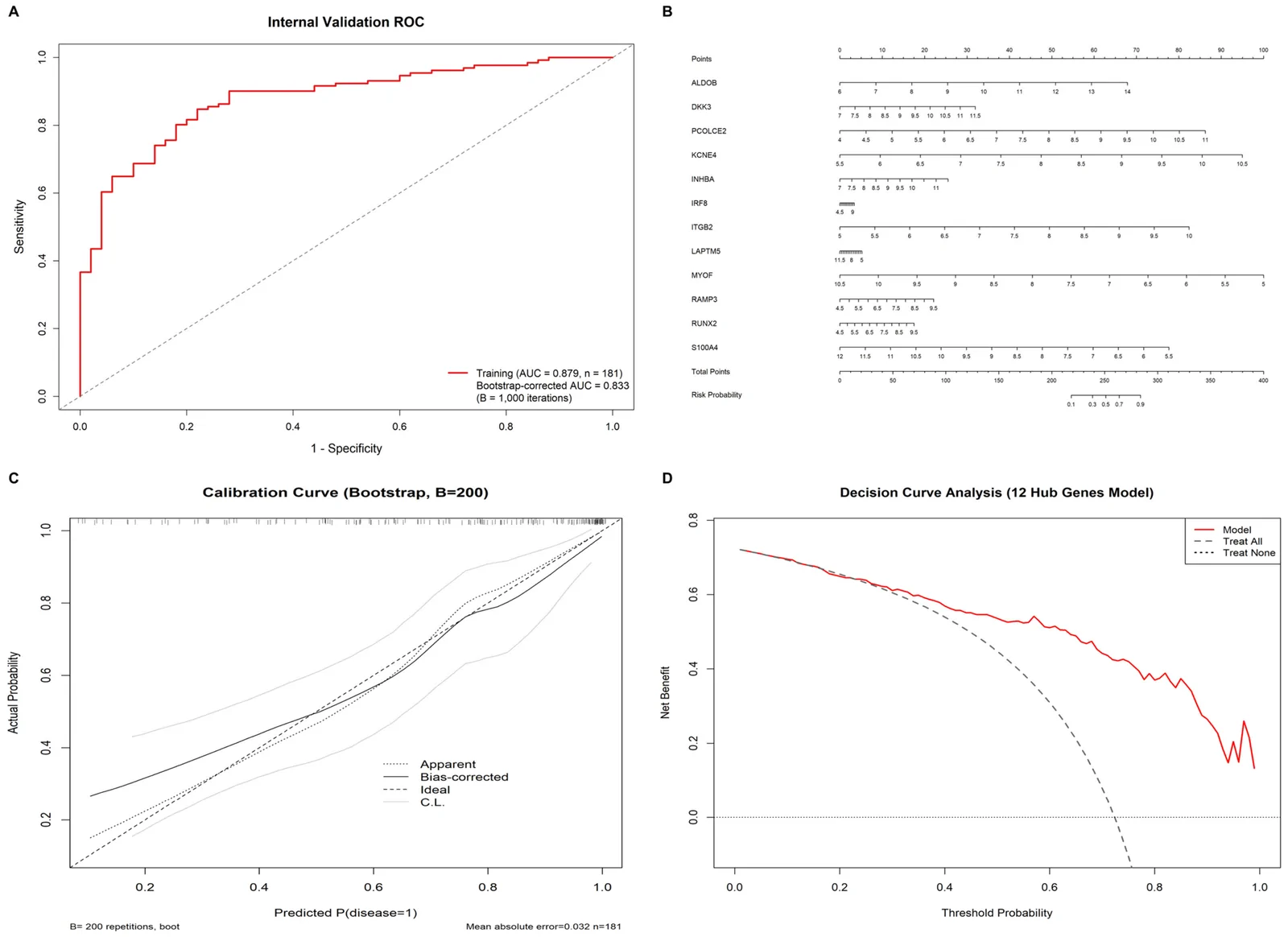
Diagnostic model performance. (**A**) Receiver operating characteristic (ROC) curve of the 12-gene logistic regression model in the discovery cohort. The apparent AUC was 0.879; bootstrap internal validation (B = 1000) yielded a corrected AUC of 0.833. (**B**) Nomogram for individualized risk prediction. (**C**) Bootstrap-corrected calibration curve (B = 200). (**D**) Decision curve analysis showing net benefit across threshold probabilities.

The logistic model based on the 12 hub genes achieved an apparent AUC of 0.879 in the training set (*n* = 181; disease 131 vs. NGT 50). Given the low EPV (4.17), 1000-iteration bootstrap validation estimated an optimism of 0.046 and an optimism-corrected AUC of 0.833 (Table 8), indicating good discrimination after accounting for overfitting (Figure 8A). Under class imbalance, the supplementary PR-AUC was 0.951 versus a random baseline of 0.724. The bootstrap-corrected calibration slope was 0.707 (Figure 8C), indicating somewhat extreme predicted probabilities and mild overfitting consistent with the low EPV; the Brier score was 0.154, indicating good overall calibration. DCA showed net benefit above the treat-all and treat-none strategies across threshold probabilities of 0.1–0.9 (Figure 8D). The nomogram (Figure 8B) facilitates clinical translation. Independent external validation was not performed owing to the scarcity of publicly available islet transcriptomic datasets with matched three-tier glucose tolerance annotations; this is acknowledged as a limitation.

**Figure 9:**
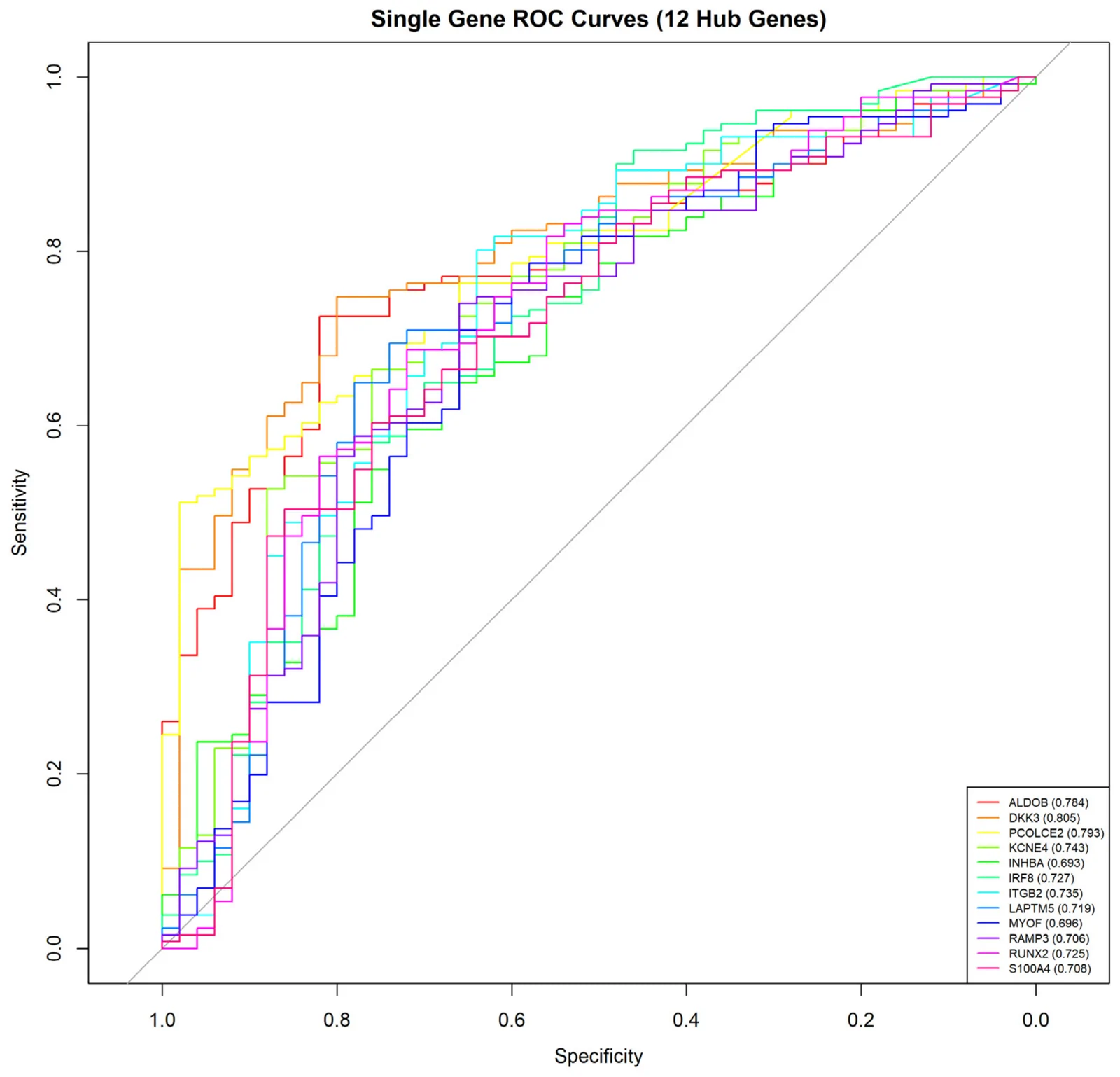
Individual diagnostic performance of the 12 hub genes. ROC curves for each of the 12 hub genes in discriminating the disease group (IGT + T2D) from NGT. AUC values are annotated for each gene. KCNE4 (AUC = 0.864), INHBA (AUC = 0.847), and PCOLCE2 (AUC = 0.843) achieved the highest individual performance. NGT, normal glucose tolerance; IGT, impaired glucose tolerance; T2D, type 2 diabetes mellitus; AUC, area under the curve; ROC, receiver operating characteristic. Individual ROC curves for the 12 hub genes. KCNE4, INHBA, and PCOLCE2 showed the best individual diagnostic performance.

In single-gene ROC analysis, KCNE4 (AUC = 0.864), INHBA (AUC = 0.847), and PCOLCE2 (AUC = 0.843) performed best (Figure 9).

### 3.9. Immune Infiltration

**Figure 10:**
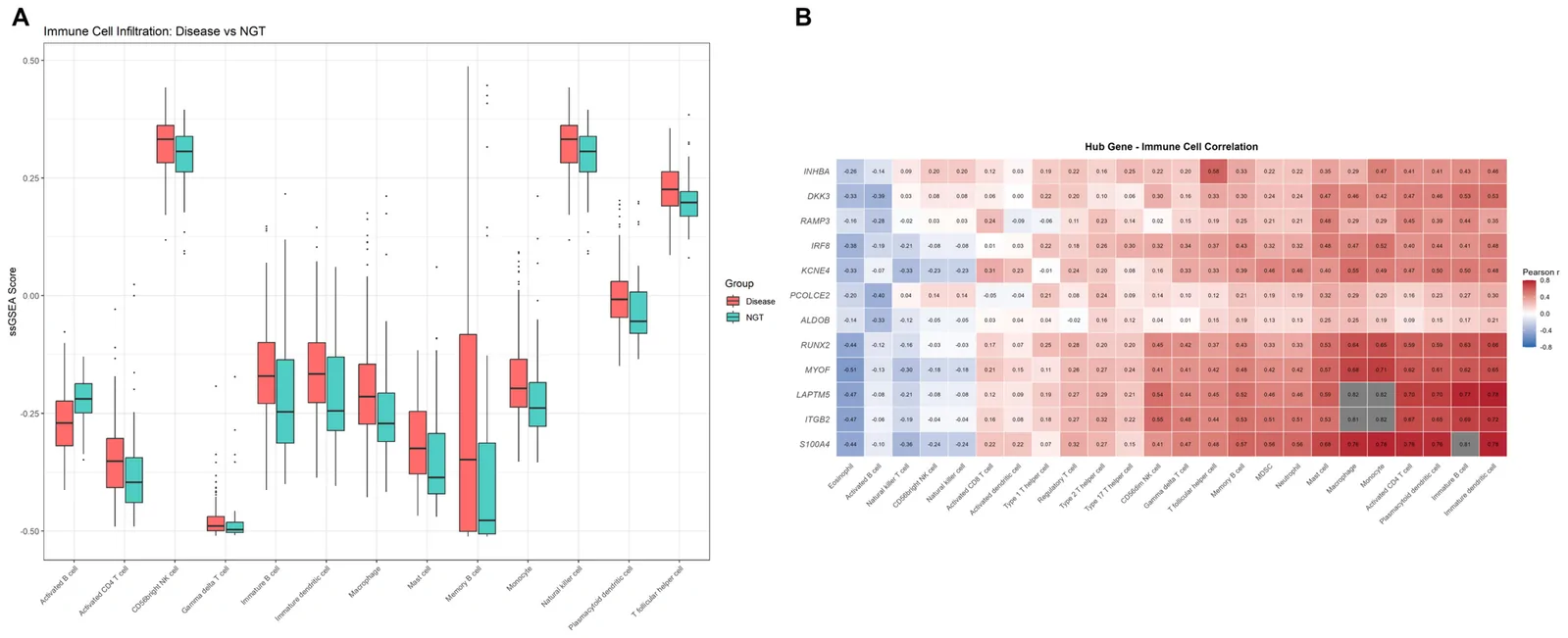
Immune infiltration landscape and hub-gene correlations. (**A**) Comparison of 24 immune cell type scores between the disease group (IGT + T2D) and NGT, quantified by ssGSEA. Cell types with significant differences (Wilcoxon test, FDR < 0.05) are highlighted; activated dendritic cells, macrophages, regulatory T cells, and γδ T cells were enriched in the disease group, whereas natural killer cells and eosinophils were depleted. (**B**) Spearman correlation heatmap between the expression of 12 hub genes and immune cell infiltration scores. Color intensity indicates correlation strength. IRF8, ITGB2, LAPTM5, and S100A4 showed strong positive correlations (r > 0.5) with multiple myeloid cell types; ALDOB showed negative correlations. ssGSEA, single-sample gene set enrichment analysis; NGT, normal glucose tolerance; IGT, impaired glucose tolerance; T2D, type 2 diabetes mellitus; FDR, false discovery rate.

ssGSEA showed that 13 of 24 immune cell types differed between the disease and NGT groups (FDR < 0.05); activated dendritic cells, macrophages, regulatory T cells, and γδ T cells increased in the disease group, whereas natural killer cells and eosinophils decreased (Figure 10A). IRF8, ITGB2, LAPTM5, and S100A4 correlated strongly with multiple myeloid cell types (r > 0.5), whereas ALDOB correlated negatively (Figure 10B). These findings suggest a correlation between low-grade islet inflammation and hub-gene expression, although the causal direction cannot be determined from cross-sectional data.

### 3.10. Single-Cell Validation of Cell-Type-Specific Hub-Gene Expression

**Figure 11:**
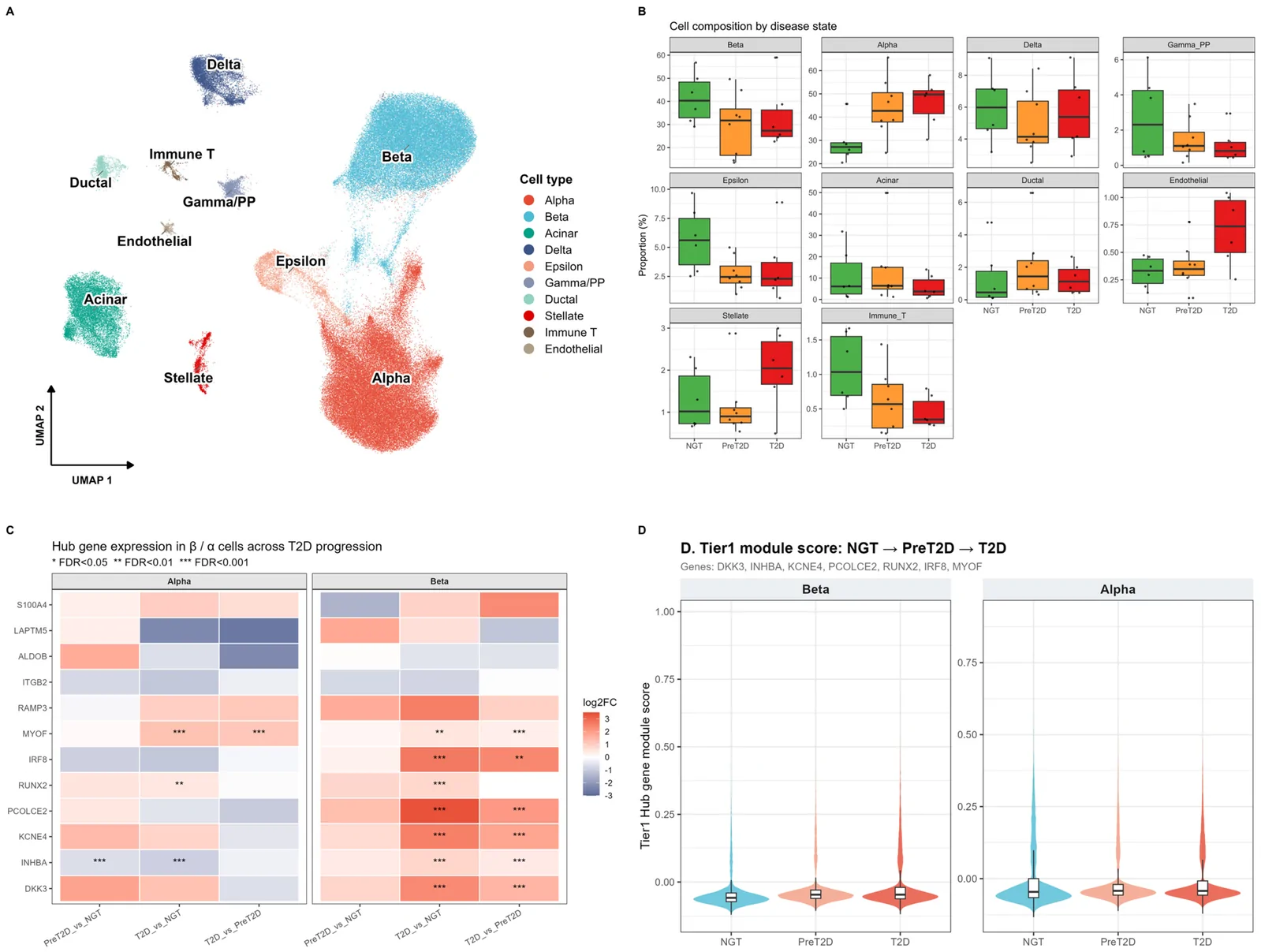
Single-cell RNA sequencing validation using GSE200044. (**A**) UMAP projection of 127,919 integrated islet cells, annotated into 10 cell types by canonical marker genes (INS, β cells; GCG, α cells; SST, δ cells; PPY, γ cells; KRT19, ductal; CPA1, acinar; PECAM1, endothelial; PDGFRA, stellate; PTPRC, immune cells). (**B**) Cell-type compositional changes across NGT, PreT2D, and T2D groups. The α-cell proportion increased progressively (28.97% → 44.26% → 46.41%; Kruskal–Wallis *p* = 0.019), whereas the β-cell proportion declined (41.33% → 29.65% → 33.24%). (**C**) Heatmap of hub-gene differential expression (log_2_FC, FDR-corrected) in β and α cells across pairwise comparisons. Seven hub genes were significantly upregulated in T2D β cells (FDR < 0.05). (**D**) Violin plots illustrating three cell-type-specific patterns invisible in bulk data: bidirectional INHBA regulation (upregulation in β cells, downregulation in α cells); early α-cell INHBA downregulation at PreT2D; and concordant MYOF and RUNX2 upregulation in both β and α cells. UMAP, uniform manifold approximation and projection; NGT, normal glucose tolerance; PreT2D, prediabetic type 2 diabetes; T2D, type 2 diabetes mellitus; FDR, false discovery rate.

After integration, 10 islet cell populations were annotated (Figure 11A), of which 43,538 β cells and 53,007 α cells were the principal analytical targets.

The α-cell proportion increased progressively from 28.97% (NGT) to 44.26% (PreT2D) and 46.41% (T2D) (Kruskal–Wallis *p* = 0.019; NGT vs. T2D Dunn *p* = 0.009), whereas the β-cell proportion showed the opposite trend (41.33% → 29.65% → 33.24%; Kruskal–Wallis *p* = 0.254). The T-lymphocyte proportion fell at T2D (1.09% vs. 0.45%; *p* = 0.041), and the endothelial proportion rose (0.32% vs. 0.71%; *p* = 0.041) (Figure 11B). The α/β imbalance is consistent with classic T2D islet pathophysiology and was already apparent at the prediabetic stage.

In β cells (*n* = 43,538), 7 of the 12 hub genes were upregulated in T2D versus NGT (FDR < 0.05; Table 9): DKK3 (log_2_FC +2.36, FDR 9.48 × 10^−35^), INHBA (+0.86, 1.21 × 10^−41^), KCNE4 (+2.55, 2.38 × 10^−31^), PCOLCE2 (+3.39, 8.52 × 10^−16^), RUNX2 (+0.82, 1.35 × 10^−8^), IRF8 (+2.68, 4.58 × 10^−4^), and MYOF (+0.54, 8.51 × 10^−3^), all directionally consistent with the bulk data and validating the cell-type origin of the bulk signal (Figure 11C). In PreT2D versus NGT, β-cell hub genes did not reach significance after FDR correction (limited by PreT2D sample size and the high-dimensional multiple-testing threshold), but early signals for RUNX2 (nominal *p* = 2.11 × 10^−5^), DKK3 (4.28 × 10^−4^), and INHBA (4.73 × 10^−4^) were directionally consistent with T2D, corroborating the progressive bulk J–T pattern.

Single-cell analysis revealed three patterns invisible in the bulk data (Figure 11D). First, INHBA was oppositely regulated: upregulated in β cells at T2D (+0.86) but downregulated in α cells (−0.91, FDR 8.59 × 10^−44^), with significant α-cell downregulation already at PreT2D (−0.64, FDR 4.58 × 10^−36^); this early α-cell signal was masked in the bulk data by net β-cell upregulation. Second, MYOF was concordantly upregulated in both β cells (+0.54) and α cells (+1.23, FDR 7.05 × 10^−54^) at T2D, with a strong late α-cell increase from PreT2D to T2D (+1.13, FDR 1.35 × 10^−115^), suggesting coordinated multi-cell activation in late T2D. Third, RUNX2 was concordantly upregulated in β cells (+0.82) and α cells (+0.48, FDR 6.46 × 10^−3^); combined with its protective MR effect (FDR 1.68 × 10^−4^), this may reflect a compensatory response to impaired islet function.

### 3.11. Pathway Enrichment Analysis

To systematically interpret the biological functions of the 74 candidate genes, GO and KEGG pathway enrichment analyses were performed using clusterProfiler.

#### 3.11.1. Go Enrichment: Ecm Remodeling as the Most Significant Biological Process

**Figure 12:**
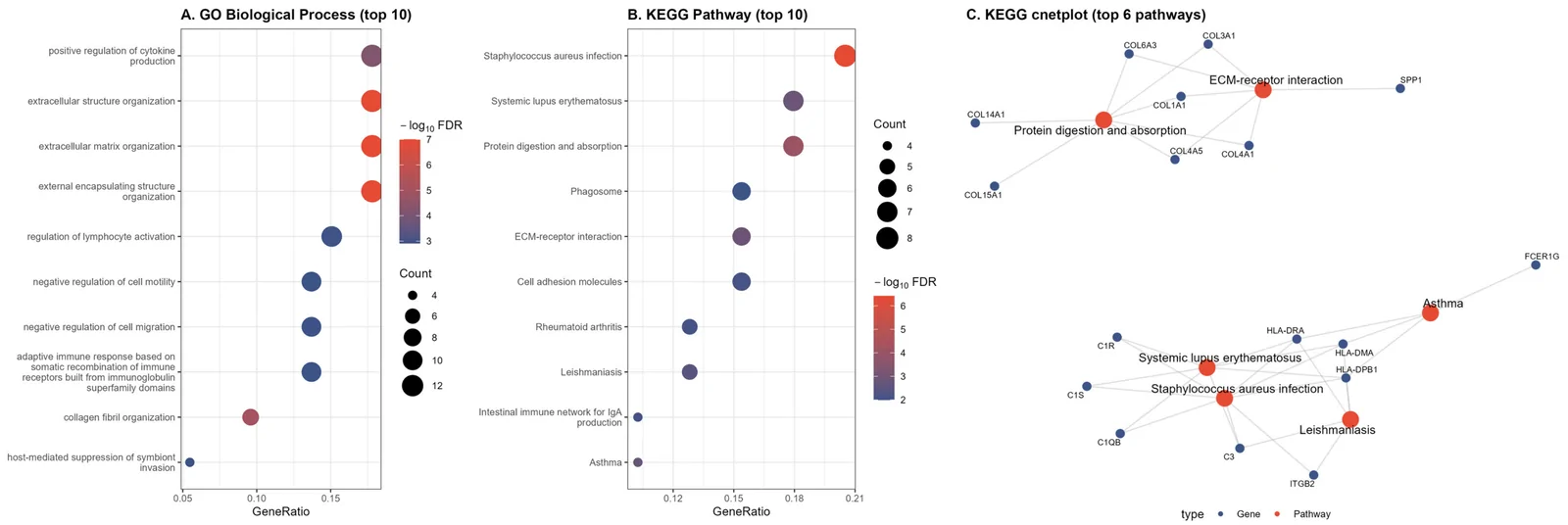
Pathway enrichment analysis of candidate and hub genes. (**A**) Dot plot of the top 10 enriched Gene Ontology biological process (GO-BP) terms for the 74 candidate genes. Dot size represents gene count and color indicates FDR value. The four most significant terms relate to extracellular matrix (ECM) remodeling; subsequent terms reflect immune regulation and cell migration. (**B**) Dot plot of 18 significantly enriched KEGG pathways (FDR < 0.05) for the candidate genes, grouped into four axes: ECM remodeling, immune activation, autoimmune-like responses, and infection response. The type 1 diabetes mellitus pathway (hsa04940) is highlighted, indicating T2D–T1D mechanistic overlap at the islet immune level. (**C**) KEGG cnetplot linking the top enriched pathways to their contributing candidate genes, illustrating the multi-gene, multi-pathway convergence on ECM remodeling and immune activation. GO, Gene Ontology; KEGG, Kyoto Encyclopedia of Genes and Genomes; ECM, extracellular matrix; FDR, false discovery rate.

At the GO BP level, the top four terms were ECM-remodeling pathways: extracellular matrix organization (13 genes, FDR 9.96 × 10^−8^), extracellular structure organization (13 genes, FDR 9.96 × 10^−8^), external encapsulating structure organization (13 genes, FDR 9.96 × 10^−8^), and collagen fibril organization (7 genes, FDR 8.35 × 10^−6^). These were followed by immune-regulation pathways (positive regulation of cytokine production, regulation of lymphocyte activation, adaptive immune response based on somatic recombination of immune receptors) and negative regulation of cell migration/motility, suggesting cooperative suppression of islet-cell migration and tissue remodeling (Figure 12A, Table 10).

#### 3.11.2. KEGG Enrichment: 18 Pathways Spanning Core T2d Pathology

KEGG enrichment identified 18 significant pathways (FDR < 0.05; Table 11) spanning four axes: ECM and cell adhesion (ECM-receptor interaction, protein digestion and absorption, cell adhesion molecules, focal adhesion); immune and complement (phagosome, antigen processing and presentation, complement and coagulation cascades); autoimmune-like responses (systemic lupus erythematosus, rheumatoid arthritis, allograft rejection, graft-versus-host disease, and the type 1 diabetes mellitus pathway hsa04940, FDR 3.44 × 10^−2^); and infection response (Staphylococcus aureus infection, the most significant pathway). The 8 co-enriched autoimmune-disease pathways shared core modules of human leukocyte antigen (HLA)-mediated antigen presentation, complement activation, and cell adhesion.

The direct hit of hsa04940 (enriched in the T2D candidate list, not only in T1D), combined with the 8 autoimmune pathways, suggests aberrant immune recognition and antigen presentation during the prediabetes-to-T2D transition, consistent with strong β-cell IRF8 upregulation in the single-cell analysis (Figure 12B,C).

The direct hit of hsa04940 suggests T2D–T1D mechanistic overlap at the islet immune level. The pathway name retains the official KEGG spelling (Roman numeral I); the disease term T1D is used in the main text. KEGG, Kyoto Encyclopedia of Genes and Genomes; ECM, extracellular matrix.

## 4. Discussion

This study identified, within a single integrated framework, a panel of 12 islet hub genes that change progressively along the NGT → IGT → T2D continuum, and elevated MYOF and RUNX2 to the level of causal evidence through MR. The methodological strengths lie at three levels: samples were stratified along a graded NGT → IGT → T2D continuum and trend-tested by the J–T method, ensuring biologically graded candidates; five complementary machine learning algorithms were combined and confirmed robust across 40 parameter combinations (Jaccard = 1.0; Table 3); and MR with Steiger directionality testing and Bayesian colocalization provided causal evidence for MYOF and RUNX2 (FDR = 1.68 × 10^−4^; Steiger *p* < 10^−36^; Table 7).

### 4.1. Biological Interpretation of the MR Causal Effects

MR analysis suggested that genetically proxied increases in MYOF (OR 0.999) and RUNX2 (OR 0.998) expression were associated with reduced T2D risk (FDR = 1.68 × 10^−4^). However, because colocalization indicated independent causal variants (MYOF PP.H3 ≈ 1.00; RUNX2 PP.H3 = 0.76), these associations may be driven by LD rather than a shared causal mechanism. The MR results therefore raise a hypothesis about blood-expression-mediated protection rather than confirming islet-level causality. The odds ratios were close to 1 because cis-eQTLs typically explain only 1–10% of expression variance; MR estimates the effect of each one-unit increase in genetically driven expression and is not directly comparable to GWAS risk-locus odds ratios. Despite small effects, both remained significant after BH-FDR correction across 8 tests. The Steiger test excluded reverse causality (ratio > 1800; *p* < 10^−36^; Table 7). However, colocalization indicated independent causal variants at both loci (MYOF PP.H3 ≈ 1.00, RUNX2 PP.H3 = 0.76), suggesting the MR effect may be influenced by LD. This warrants cautious interpretation: eQTLGen reflects whole blood rather than islet tissue, and inter-tissue differences in cis-regulation may cause loss of colocalization; allelic heterogeneity may also allow multiple functional variants to act independently. Reassessment is warranted once islet-specific eQTL resources become available. Taken together, the MR evidence reaches the level of a plausible hypothesis rather than a validated causal claim; the Abstract, Conclusions, and interpretive language have been aligned accordingly.

MYOF and RUNX2 were upregulated in the disease group (log_2_FC +0.55 and +0.59), whereas MR indicated protective effects of high expression. These directions are consistent rather than contradictory: disease-group upregulation can be interpreted as a compensatory stress response in which protective genes rise under metabolic stress to maintain islet homeostasis, but with capacity insufficient to halt progression. MYOF participates in membrane repair and vesicle trafficking, potentially supporting β-cell insulin-vesicle release; RUNX2, a matrix-remodeling transcription factor, showed coordinated β/α-cell upregulation consistent with collagen fibril organization enrichment (FDR 8.35 × 10^−6^).

### 4.2. Integration of Three Pathophysiological Axes

The enrichment results and single-cell validation together support an integrated “ECM remodeling–immune activation–autoimmune-like response” three-axis mechanism. For the ECM axis, the candidate genes were enriched in 4 ECM-related GO BP pathways (FDR < 10^−7^) and 4 KEGG pathways. Through single-cell analysis, we localized this signal to specific cell types—PCOLCE2 strongly upregulated in β cells (+3.39), RUNX2 coordinately upregulated in β/α cells, and DKK3 upregulated in β cells (+2.36)—forming an evidence chain from bulk signal to cell-type localization to pathway confirmation. This complements, at the molecular annotation level, the β-cell drift toward a stress/dedifferentiation trajectory reported by Wigger et al. through multi-omics profiling of living islet donors [14].

For the immune and autoimmune-like axis, the candidate genes were enriched in 8 autoimmune-disease KEGG pathways (including hsa04940) and complement and coagulation cascades, consistent with the inflammatory-disease thesis of Donath et al. [10], the islet-macrophage review of Eguchi et al. [38], and the β-cell stress-pathway overlap proposed by Eizirik et al. [12]. The hsa04940 enrichment involved only 3 genes at a marginal FDR (0.034) and more likely reflects upregulation of HLA antigen-presentation molecules due to islet immune-cell infiltration rather than intrinsic β-cell autoimmunity; accordingly, the T2D–T1D overlap should be framed as shared pathways at the level of islet immune infiltration. The unique contributions are twofold: the direct hit of hsa04940 strengthens the islet-immune overlap hypothesis with omics evidence in one framework, and the combination of increased dendritic cells, macrophages, regulatory T cells, and γδ T cells with strong β-cell IRF8 upregulation localizes the bulk pathway signal to specific cell types and a transcription-factor hub. This provides a biological basis for immune-intervention strategies at the prediabetic stage, complementing the prevention effects reported by Knowler et al. [4].

For the cell-migration and composition axis, enrichment of negative regulation of cell migration and motility, combined with the α/β imbalance (α cells 28.97% → 46.41%; Kruskal–Wallis *p* = 0.019), suggests that the candidate genes participate in T2D pathology by regulating intra-islet cell migration and subpopulation redistribution—an abnormality already apparent at PreT2D, validating at single-cell resolution the clinical observation by Halban et al. [39] that islet composition abnormalities precede overt hyperglycemia.

### 4.3. Kcne4 as the Earliest Candidate Marker

KCNE4 encodes a voltage-gated potassium channel auxiliary subunit. Its single-gene ROC AUC (0.864) ranked first, and it reached 94.0% of its T2D effect size at IGT (log_2_FC 0.580 vs. 0.617; nominal *p* = 1.75 × 10^−4^), upregulated ahead of most metabolism- and inflammation-related genes. Given that no transcriptome-wide DEGs reached the threshold at IGT (Supplementary File: Figure S1), the early KCNE4 change is notable. Mechanistically, KCNE4 couples with channels such as KCNQ1 to regulate β-cell resting potential and first-phase insulin secretion; Hudish et al. noted that altered potassium currents are early events along the metabolic syndrome–T2D axis [40], and our IGT-stage transcriptomic data support this. Single-cell analysis confirmed strong β-cell KCNE4 upregulation at T2D (+2.55), localizing the early change as a β-cell-specific event. Reports of KCNE4 in T2D islets are limited, and its early prediabetic alteration has not been systematically characterized, positioning it as a candidate biomarker for early warning and risk stratification, pending validation in larger independent prediabetes cohorts and aligned with consensus emphasis on early identification of high-risk populations [41].

### 4.4. Bidirectional Inhba Regulation

INHBA encodes the inhibin βA subunit of activin A. It was upregulated in β cells at T2D (+0.86) but downregulated in α cells (−0.91), with significant α-cell downregulation already at PreT2D (−0.64). This cell-type-specific opposite regulation has not been clearly reported in prior islet T2D literature. Mechanistically, β-cell activin A may promote apoptosis and dedifferentiation, whereas reduced α-cell activin signaling may relieve inhibition of glucagon secretion [42], together driving insulin/glucagon imbalance. In the bulk transcriptome, the β-cell upregulation and α-cell downregulation offset each other, producing an illusory net effect that masked the underlying biology—corroborating the argument of Wigger et al. that cell-type heterogeneity is central to islet T2D pathology [14] and demonstrating the necessity of single-cell deconstruction, while suggesting cell-type-specific targets such as selective activin-signaling modulators.

### 4.5. Model Positioning and Comparison with Prior Studies

After bootstrap validation, the 12-gene model achieved an AUC of 0.833 with a PR-AUC of 0.951 and good calibration (Table 8). Because the EPV (4.17) was below the recommended threshold, the model is positioned as a classification-discrimination tool rather than a clinical prediction instrument, pending independent-cohort validation. Compared with prior work, Solimena et al. [13] identified functional-decline genes by islet RNA-seq but lacked causal inference, whereas we elevated the evidence to the causal level via MR and excluded reverse causality. Wigger et al. [14] performed longitudinal metabolome–transcriptome profiling (*n* = 45) without multiple-testing correction, whereas we integrated two islet datasets (*n* = 181, including 56 independent IGT samples) with BH-FDR correction across 8 MR tests. Neither prior study employed our machine learning cross-validation and single-cell deconvolution strategies.

### 4.6. Limitations

This study has several limitations. First, both training datasets were cross-sectional (*n* = 181); the J–T test provides graded evidence, but establishing causal trajectories requires prospective cohorts. Second, eQTLGen instruments reflect whole-blood rather than islet expression; colocalization indicated independent causal variants (PP.H3 > 0.76), so LD confounding cannot be excluded. Both exposure and outcome data were restricted to European populations, and the single-causal-variant assumption of coloc may be violated at multi-signal loci (addressable by SuSiE-coloc in future work). Third, MYOF and RUNX2 showed parameter sensitivity in feature selection, though their causal associations were independently supported by MR. Fourth, the diagnostic model EPV was below threshold, and no independent external validation was performed; the bootstrap-corrected AUC of 0.833 represents an optimism-adjusted estimate pending confirmation in external cohorts. Fifth, single-cell validation relied on one dataset (GSE200044), and progressive prediabetic evidence rests on the bulk J–T test. Sixth, the MR-implicated protective roles of MYOF and RUNX2 lack functional experimental validation. Seventh, individual-level clinical metadata (age, BMI, sex) were available only for GSE76895 (83 of 181 samples); although no demographic differences were observed among groups, the absence of matched metadata for GSE164416 precludes formal assessment of between-dataset confounding beyond batch correction.

## 5. Conclusions

We integrated 181 islet transcriptomes, five machine learning algorithms, MR causal inference, the J–T trend test, bootstrap validation, single-cell validation, and pathway enrichment to identify 12 hub genes that change progressively along the NGT → IGT → T2D axis. All 12 increased monotonically across the three groups (J–T test, 12/12 at the permutation floor of *p* ≤ 10^−4^, ranking in the top 0.13–2.69% of 11,948 transcriptome-wide tests). KCNE4 reached 94% of its T2D effect size at IGT, marking it as the earliest-altered candidate at the prediabetic stage. MR suggested protective associations for MYOF and RUNX2 (FDR = 1.68 × 10^−4^) with a supported expression-to-disease direction (Steiger *p* < 10^−36^); however, colocalization indicated independent causal variants at both loci (PP.H3 > 0.76), substantially weakening the causal interpretation. These MR findings therefore constitute a hypothesis requiring validation with islet-specific eQTL data once available. Combined with their disease-group upregulation, these two genes may represent endogenous compensatory molecules. The 12-gene logistic model achieved a bootstrap-corrected AUC of 0.833 and PR-AUC of 0.951. Single-cell validation confirmed β-cell upregulation of 7 hub genes and revealed bulk-invisible signals, including bidirectional INHBA regulation and early α-cell INHBA downregulation. Enrichment revealed three mechanistic axes, with the direct hit of hsa04940 suggesting T2D–T1D overlap at the islet immune level. This multilayered evidence chain—from bulk signal to cell-type mechanism to causal validation—identifies KCNE4 as an early prediabetic marker and MYOF/RUNX2 as compensatory protective targets, laying a foundation for precision-intervention strategies. The model should be positioned as an islet-tissue-specific auxiliary diagnostic tool; future studies should explore surrogate markers (peripheral blood, circulating microRNAs, exosomes) and validate performance in independent islet and multi-population cohorts.

## Figures and Tables

**Table 1:** Composition of study samples. (**A**). Dataset composition (**B**). Donor characteristics (GSE76895, *n* = 83) †.

(**A**)											
**Dataset**	**Platform**		**Tissue**		**NGT**	**IGT**		**T2D**	**Total**		**Use**
GSE76895	GPL570		Islet		32	15		36	83		Training
GSE164416	GPL16791		Islet		18	41		39	98		Training
Training total	—		—		50	56		75	181		—
GSE200044	10× Genomics		Islet (single cell)		31,884 ^a^	46,704 ^a^		49,331 ^a^	127,919 ^a^		Single-cell validation
(**B**)											
**Characteristic**		**NGT (*n* = 32)**		**IGT (*n* = 15)**			**T2D (*n* = 36)**			***p***	
Age (years)		60.0 ± 13.8		62.8 ± 12.6			65.8 ± 12.2			0.190	
BMI (kg/m^2^)		24.9 ± 3.4		25.7 ± 3.5			25.8 ± 5.0			0.682	
Sex, male *n* (%)		16 (50.0%)		9 (60.0%)			23 (63.9%)			0.503	
Fasting glucose (mmol/L)		5.3 ± 0.6		5.3 ± 0.4			8.0 ± 2.7			1.9 × 10^−7^	

^a^ Cell counts. † GSE164416 did not deposit individual-level clinical metadata in GEO; characteristics are from GSE76895 only. Data shown as mean ± SD or *n* (%). *p*-values from one-way ANOVA (continuous) or χ^2^ test (categorical). The glucose tolerance group distribution differed significantly between datasets (χ^2^ *p* = 6 × 10^−4^); dataset origin was included as a covariate in downstream analyses. HbA1c and HOMA-IR were not available in either dataset.

**Table 2:** Characteristics of the 12 hub genes.

Gene	Cytoband	Functional Annotation	log_2_FC (Disease vs. NGT)	FDR
ALDOB	9q31.1	Glycolytic aldolase	+1.32	<0.001
DKK3	11p15.3	Wnt signaling inhibitor	+0.95	<0.001
PCOLCE2	3q21.3	Collagen maturation cofactor	+1.18	<0.001
KCNE4	2q36.1	Potassium channel auxiliary subunit	+0.61	<0.001
INHBA	7p14.1	Inhibin βA subunit	+0.55	<0.001
IRF8	16q24.1	Immune-regulatory transcription factor	+0.78	<0.001
ITGB2	21q22.3	Integrin β2	+0.61	<0.001
LAPTM5	1p34.3	Lysosomal transmembrane protein	+0.69	<0.001
MYOF	10q23.33	Membrane-repair myoferlin	+0.55	<0.001
RAMP3	7p13	Receptor activity-modifying protein	+0.56	<0.001
RUNX2	6p21.1	Matrix-remodeling transcription factor	+0.59	<0.001
S100A4	1q21.3	Calcium-binding protein	+0.69	<0.001

**Table 3:** Threshold sensitivity: individual stability of hub genes.

Gene	Times Selected	Combinations	Frequency (%)	Stability Class
ALDOB	40	40	100.0	Highly stable
DKK3	40	40	100.0	Highly stable
PCOLCE2	40	40	100.0	Highly stable
KCNE4	30	40	75.0	Moderately stable
ITGB2	18	40	45.0	Threshold-sensitive
RUNX2	17	40	42.5	Threshold-sensitive
LAPTM5	17	40	42.5	Threshold-sensitive
MYOF	15	40	37.5	Threshold-sensitive
INHBA	15	40	37.5	Threshold-sensitive
IRF8	15	40	37.5	Threshold-sensitive
RAMP3	15	40	37.5	Threshold-sensitive
S100A4	15	40	37.5	Threshold-sensitive

**Table 4:** Differential expression of hub genes at IGT and T2D stages.

Gene	IGT log_2_FC	IGT *p*	IGT FDR	T2D log_2_FC	T2D *p*	T2D FDR	IGT/T2D Ratio (%)	Stage Pattern
KCNE4	0.580	1.75 × 10^−4^	0.283	0.617	1.45 × 10^−6^	7.53 × 10^−5^	94.0	Early progressive
S100A4	0.534	0.0273	0.458	0.800	1.87 × 10^−5^	4.89 × 10^−4^	66.8	Progressive
RAMP3	0.426	0.00388	0.333	0.662	1.05 × 10^−6^	6.19 × 10^−5^	64.4	Progressive
INHBA	0.401	0.0114	0.364	0.667	1.28 × 10^−6^	7.02 × 10^−5^	60.1	Progressive
LAPTM5	0.474	0.0279	0.462	0.901	2.09 × 10^−6^	1.00 × 10^−4^	52.6	Progressive
ITGB2	0.390	0.0217	0.445	0.795	2.81 × 10^−6^	1.25 × 10^−4^	49.1	Progressive
RUNX2	0.332	0.0630	0.564	0.788	2.02 × 10^−7^	1.68 × 10^−5^	42.1	Progressive
MYOF	0.278	0.115	0.657	0.762	5.41 × 10^−7^	3.76 × 10^−5^	36.5	T2D-dominant
DKK3	0.448	0.00933	0.355	1.272	2.21 × 10^−20^	1.32 × 10^−16^	35.2	T2D-dominant
IRF8	0.184	0.285	0.805	1.120	6.87 × 10^−12^	3.28 × 10^−9^	16.4	T2D-dominant
ALDOB	0.334	0.0689	0.576	2.053	5.63 × 10^−18^	1.12 × 10^−14^	16.3	T2D-dominant
PCOLCE2	0.172	0.200	0.750	1.794	2.69 × 10^−19^	8.05 × 10^−16^	9.6	T2D-dominant

IGT *n* = 56, NGT *n* = 50, T2D *n* = 75; thresholds |log_2_FC| > 0.5 and FDR < 0.05; all genes directionally consistent (upregulated). Early progressive: nominal *p* < 0.05 at IGT with effect size ≥ 80% of T2D; progressive: nominal *p* < 0.05 at IGT; T2D-dominant: nominal *p* ≥ 0.05 at IGT. IGT/T2D ratio = log_2_FC(IGT)/log_2_FC(T2D) × 100%.

**Table 5:** Jonckheere–Terpstra trend test for the 12 hub genes.

Gene	NGT Median	IGT Median	T2D Median	J–T Statistic	*p*_Trend	FDR (BH)	Bonferroni	Significant
ALDOB	7.21	7.96	8.42	8791.0	<10^−4^	1.09 × 10^−4^	1.2 × 10^−3^	Yes
DKK3	8.78	9.23	9.85	8525.0	<10^−4^	1.09 × 10^−4^	1.2 × 10^−3^	Yes
PCOLCE2	6.12	6.84	7.62	8905.5	<10^−4^	1.09 × 10^−4^	1.2 × 10^−3^	Yes
KCNE4	5.34	5.98	6.43	7213.0	<10^−4^	1.09 × 10^−4^	1.2 × 10^−3^	Yes
INHBA	6.21	6.74	7.05	7211.0	<10^−4^	1.09 × 10^−4^	1.2 × 10^−3^	Yes
IRF8	4.82	5.46	6.13	8172.0	<10^−4^	1.09 × 10^−4^	1.2 × 10^−3^	Yes
ITGB2	5.12	5.78	6.27	7491.5	<10^−4^	1.09 × 10^−4^	1.2 × 10^−3^	Yes
MYOF	7.34	7.71	8.10	7482.0	<10^−4^	1.09 × 10^−4^	1.2 × 10^−3^	Yes
RAMP3	5.65	6.13	6.54	7137.0	<10^−4^	1.09 × 10^−4^	1.2 × 10^−3^	Yes
RUNX2	4.98	5.62	6.05	7560.5	<10^−4^	1.09 × 10^−4^	1.2 × 10^−3^	Yes
S100A4	5.43	6.11	6.65	7075.0	<10^−4^	1.09 × 10^−4^	1.2 × 10^−3^	Yes
LAPTM5	6.43	6.78	7.45	7260.5	≤2 × 10^−4^	2.0 × 10^−4^	2.4 × 10^−3^	Yes

**Table 6:** Mendelian randomization results for the 8 candidate genes.

Gene	Instruments	Method	OR	95% CI	F	*p*	FDR (BH)	Bonferroni	Direction
MYOF	2	IVW	0.999	0.999–1.000	>29.7	2.79 × 10^−5^	1.68 × 10^−4^	2.23 × 10^−4^	Protective
RUNX2	2	IVW	0.998	0.998–0.999	>29.7	4.20 × 10^−5^	1.68 × 10^−4^	3.36 × 10^−4^	Protective
RAMP3	2	IVW	0.999	0.998–1.000	>29.7	0.116	0.309	0.928	—
DKK3	1	Wald	1.012	0.987–1.039	>29.7	0.349	0.698	1.000	—
ITGB2	1	Wald	1.009	0.986–1.032	>29.7	0.453	0.725	1.000	—
LAPTM5	1	Wald	1.007	0.965–1.050	>29.7	0.757	0.792	1.000	—
PCOLCE2	1	Wald	1.027	0.862–1.224	>29.7	0.764	0.792	1.000	—
ALDOB	1	Wald	0.989	0.914–1.071	>29.7	0.792	0.792	1.000	—

Exposure: eQTLGen cis-eQTLs (*p* < 5 × 10^−8^, LD r^2^ < 0.001, distance > 10,000 kb); outcome: Suzuki et al. 2024 T2D GWAS (European ancestry) [23]. All instruments had F = Z^2^ > 29.7. FDR correction used BH (*n* = 8). OR is per one-unit increase in standardized cis-eQTL effect on T2D risk. Single-instrument genes used the Wald ratio and could not undergo MR-Egger or Q tests. KCNE4, INHBA, IRF8, and S100A4 lacked instruments. OR, odds ratio; CI, confidence interval; IVW, inverse-variance weighted.

**Table 7:** Steiger directionality and colocalization for MR-significant genes.

Gene	Instrument	R^2^_Exposure	R^2^_Outcome	R^2^ Ratio	Steiger Z	Steiger *p*	Direction	nSNPs	PP.H3	PP.H4	Conclusion
MYOF	rs11187158	1.28 × 10^−3^	2.43 × 10^−5^	52.7	3.39	6.96 × 10^−4^	Expr → T2D	—	—	—	—
MYOF	rs11594445	4.39 × 10^−2^	1.15 × 10^−7^	381,739	23.98	4.55 × 10^−127^	Expr → T2D	—	—	—	—
MYOF (combined)	—	4.52 × 10^−2^	2.44 × 10^−5^	1855	23.49	5.41 × 10^−122^	Expr → T2D	6306	≈1.00	≈0	Independent variants
RUNX2	rs3799973	1.08 × 10^−2^	2.25 × 10^−5^	480	12.53	5.31 × 10^−36^	Expr → T2D	—	—	—	—
RUNX2	rs1200428	9.44 × 10^−2^	3.05 × 10^−7^	309,508	39.89	<10^−300^	Expr → T2D	—	—	—	—
RUNX2 (combined)	—	1.05 × 10^−1^	2.28 × 10^−5^	4612	41.75	<10^−300^	Expr → T2D	6275	0.76	0.0006	Independent variants

R^2^_exposure = Z^2^/(Z^2^ + N), variance explained for expression (eQTLGen whole blood, N ≤ 31,684); R^2^_outcome = (Beta/SE)^2^/N_eff, variance explained for T2D (Suzuki et al. 2024 [23], N_eff ≤ 692,668). R^2^ ratio = R^2^_exposure/R^2^_outcome; ratio ≫ 1 supports gene-expression-to-T2D. PP.H3 > 0.5 indicates independent causal variants; PP.H4 ≥ 0.75 indicates a shared variant. Default priors p1 = p2 = 1 × 10^−4^, p12 = 1 × 10^−5^. Colocalization is reported per gene (combined row). Analyses were restricted to BH-FDR-significant genes (FDR < 0.05).

**Table 8:** Bootstrap internal validation of the diagnostic model.

Metric	Value	Interpretation
Apparent AUC	0.879	Apparent discrimination
Optimism	0.046	Mean train–test gap
Corrected AUC	0.833	Discrimination after removing overfitting
PR-AUC	0.951	Precision-recall (baseline 0.724)
Calibration slope	0.707	Ideal = 1.0; mild overfitting
Brier score	0.154	Closer to 0 is better
Mean calibration error	0.033	Average calibration error
EPV	4.17	Below recommended threshold of 10

Bootstrap B = 1000; calibration curve from 200 resamples. AUC, area under the curve; PR-AUC, precision-recall AUC; EPV, events per variable.

**Table 9:** Single-cell DEG validation of hub genes in β and α cells.

Gene	β PreT2D/NGT	β T2D/NGT	β T2D/PreT2D	α PreT2D/NGT	α T2D/NGT	α T2D/PreT2D
DKK3	ns	**+2.36** (9.5 × 10^−35^)	**+1.51** (4.7 × 10^−20^)	ns	ns	ns
INHBA	ns	**+0.86** (1.2 × 10^−41^)	**+0.44** (6.2 × 10^−22^)	**−0.64** (4.6 × 10^−36^)	**−0.91** (8.6 × 10^−44^)	ns
KCNE4	ns	**+2.55** (2.4 × 10^−31^)	**+1.83** (8.7 × 10^−20^)	ns	ns	ns
PCOLCE2	ns	**+3.39** (8.5 × 10^−16^)	**+2.08** (3.6 × 10^−13^)	ns	ns	ns
RUNX2	ns	**+0.82** (1.4 × 10^−8^)	ns	ns	**+0.48** (6.5 × 10^−3^)	ns
IRF8	ns	**+2.68** (4.6 × 10^−4^)	**+2.41** (3.8 × 10^−3^)	ns	ns	ns
MYOF	ns	**+0.54** (8.5 × 10^−3^)	**+0.35** (1.4 × 10^−7^)	ns	**+1.23** (7.1 × 10^−54^)	**+1.13** (1.4 × 10^−115^)
ALDOB	ns	ns	ns	ns	ns	ns
RAMP3	ns	ns	ns	ns	ns	ns
ITGB2	ns	ns	ns	ns	ns	ns
LAPTM5	ns	ns	ns	ns	ns	ns
S100A4	ns	ns	ns	ns	ns	ns

Values are log_2_FC (FDR); β cells *n* = 43,538, α cells *n* = 53,007; BH correction (38,107 genes per comparison); bold indicates FDR < 0.05; ns indicates FDR ≥ 0.05; NGT *n* = 31,884, PreT2D *n* = 46,704, T2D *n* = 49,331. DEG, differentially expressed gene.

**Table 10:** Top 10 enriched GO biological processes for candidate genes.

Pathway	Gene Count	*p*	FDR
Extracellular matrix organization	13	4.65 × 10^−11^	9.96 × 10^−8^
Extracellular structure organization	13	4.86 × 10^−11^	9.96 × 10^−8^
External encapsulating structure organization	13	5.08 × 10^−11^	9.96 × 10^−8^
Collagen fibril organization	7	5.68 × 10^−9^	8.35 × 10^−6^
Positive regulation of cytokine production	13	5.46 × 10^−8^	6.42 × 10^−5^
Regulation of lymphocyte activation	11	1.28 × 10^−6^	1.16 × 10^−3^
Negative regulation of cell migration	10	1.38 × 10^−6^	1.16 × 10^−3^
Adaptive immune response (immune-receptor recombination)	10	1.85 × 10^−6^	1.22 × 10^−3^
Negative regulation of cell motility	10	1.98 × 10^−6^	1.22 × 10^−3^
Host-mediated suppression of symbiont invasion	4	2.08 × 10^−6^	1.22 × 10^−3^

GO, Gene Ontology; FDR, false discovery rate (Benjamini–Hochberg correction); *p*-values are from a two-sided hypergeometric test.

**Table 11:** KEGG pathway enrichment of candidate genes (18 pathways).

ID	Pathway	Gene Count	FDR	Axis
hsa05150	Staphylococcus aureus infection	8	3.84 × 10^−7^	Infection response
hsa04974	Protein digestion and absorption	7	8.79 × 10^−5^	ECM remodeling
hsa04512	ECM-receptor interaction	6	1.27 × 10^−3^	ECM remodeling
hsa05322	Systemic lupus erythematosus	7	1.41 × 10^−3^	Autoimmune
hsa05310	Asthma	4	1.79 × 10^−3^	Immune activation
hsa05140	Leishmaniasis	5	3.72 × 10^−3^	Infection response
hsa04514	Cell adhesion molecules	6	6.78 × 10^−3^	ECM remodeling
hsa04672	Intestinal immune network for IgA production	4	7.29 × 10^−3^	Immune activation
hsa05323	Rheumatoid arthritis	5	7.52 × 10^−3^	Autoimmune
hsa04145	Phagosome	6	1.01 × 10^−2^	Immune activation
hsa05146	Amoebiasis	5	1.25 × 10^−2^	Infection response
hsa04610	Complement and coagulation cascades	4	1.90 × 10^−2^	Immune activation
hsa05416	Viral myocarditis	4	1.90 × 10^−2^	Autoimmune
hsa04612	Antigen processing and presentation	4	2.40 × 10^−2^	Immune activation
hsa05330	Allograft rejection	3	2.57 × 10^−2^	Autoimmune
hsa04510	Focal adhesion	6	2.57 × 10^−2^	ECM remodeling
hsa05332	Graft-versus-host disease	3	3.17 × 10^−2^	Autoimmune
hsa04940	Type I diabetes mellitus	3	3.44 × 10^−2^	Autoimmune

## Data Availability

The transcriptomic datasets analyzed in this study are publicly available from the NCBI Gene Expression Omnibus (GEO): GSE76895 (https://www.ncbi.nlm.nih.gov/geo/query/acc.cgi?acc=GSE76895, accessed on 22 May 2026), GSE164416 (https://www.ncbi.nlm.nih.gov/geo/query/acc.cgi?acc=GSE164416, accessed on 22 May 2026), and GSE200044 (https://www.ncbi.nlm.nih.gov/geo/query/acc.cgi?acc=GSE200044, accessed on 22 May 2026). The cis-eQTL summary statistics used for Mendelian randomization are available from the eQTLGen Consortium (https://www.eqtlgen.org/, accessed on 22 May 2026). The type 2 diabetes GWAS summary statistics are available from Suzuki et al. [23] via the NHGRI-EBI GWAS Catalog (accession GCST90132184). The analysis code supporting the conclusions of this article is available from the corresponding author upon reasonable request.

## Supplementary Materials

The following supporting information can be downloaded at: https://www.mdpi.com/article/10.3390/biomedicines14081850/s1, Table S1: complete machine learning threshold sensitivity results (40 parameter combinations); Table S2: instrumental-variable details for MYOF and RUNX2; Table S3: colocalization sensitivity to the p12 prior (1 × 10^−6^ to 1 × 10^−4^); Figure S1: volcano plot of IGT versus NGT differential expression (no significant DEGs); Figure S2: transcriptome-wide Jonckheere–Terpstra trend test *p*-value distribution (11,948 genes); Figure S3: transcriptome-wide J–T test gene ranking plot with the 12 hub genes highlighted.

## Abbreviations

T2D: Type 2 diabetes mellitus; T1D: Type 1 diabetes mellitus; NGT: Normal glucose tolerance; IGT: Impaired glucose tolerance; PreT2D: Prediabetic type 2 diabetes; IFG: Impaired fasting glucose; WGCNA: Weighted gene co-expression network analysis; MR: Mendelian randomization; eQTL: Expression quantitative trait locus; cis-eQTL: cis-expression quantitative trait locus; GWAS: Genome-wide association study; LD: Linkage disequilibrium; SNP: Single nucleotide polymorphism; LASSO: Least absolute shrinkage and selection operator; SVM-RFE: Support vector machine recursive feature elimination; IVW: Inverse-variance weighted; J–T: Jonckheere–Terpstra; FDR: False discovery rate; BH: Benjamini–Hochberg; OR: Odds ratio; CI: Confidence interval; ssGSEA: Single-sample gene set enrichment analysis; ROC: Receiver operating characteristic; AUC: Area under the curve; PR-AUC: Precision-recall area under the curve; DCA: Decision curve analysis; EPV: Events per variable; DEG: Differentially expressed gene; ECM: Extracellular matrix; GO: Gene Ontology; BP: Biological process; KEGG: Kyoto Encyclopedia of Genes and Genomes; HLA: Human leukocyte antigen; UMAP: Uniform manifold approximation and projection; PCA: Principal component analysis; scRNA-seq: Single-cell RNA sequencing; PP: Posterior probability.
