# Supplementary material for: Identification of Progressive Islet Biomarkers for Type 2 Diabetes by Integrated Transcriptomics and Mendelian Randomization: Alterations Spanning the Normal Glucose Tolerance–Impaired Glucose Tolerance–Type 2 Diabetes Continuum
Source: Biomedicines. 2026 Aug 18;14(8):1850. doi: 10.3390/biomedicines14081850 (PMC13510233; doi:10.3390/biomedicines14081850)
Supplement: Supplementary file 1 [file biomedicines-14-01850-s001.zip › biomedicines-4458437-supplementary.pdf]

All Supplementary Tables (based on correct R output)

Supplementary Table S1: Sensitivity Analysis of Coloc p12 Prior

Supplementary Table S1. Sensitivity of colocalization results to p12 prior

Gene	p12	PP.H3	PP.H4	Conclusion
MYOF	1×10^{-6}	1.0000	0.0000	Independent
MYOF	5×10^{-6}	1.0000	0.0000	Independent
MYOF	1×10^{-5}	1.0000	0.0000	Independent
MYOF	5×10^{-5}	1.0000	0.0000	Independent
MYOF	1×10^{-4}	1.0000	0.0000	Independent
RUNX2	1×10^{-6}	0.0005	0.0003	Inconclusive
RUNX2	5×10^{-6}	0.0005	0.0013	Inconclusive
RUNX2	1×10^{-5}	0.0005	0.0026	Inconclusive
RUNX2	5×10^{-5}	0.0005	0.0127	Inconclusive
RUNX2	1×10^{-4}	0.0005	0.0251	Inconclusive

Note: The default priors are p1 = p2 = 1×10^{-4} and p12 = 1×10^{-5} (bold rows). MYOF represents an independent causal variant under all priors (PP.H3 ≈ 1.00). RUNX2 had PP.H3 = 0.76 (independent) under the default prior, but in the sensitivity analysis PP.H3 dropped to < 0.001 while PP.H4 remained < 0.03, so it was classified as Inconclusive rather than Independent—this is because PP.H1 (eQTL association only) = 0.24 absorbed most of the probability in the sensitivity recalculation. coloc package v5.2.3; eQTL: eQTLGen Consortium (whole blood, N ≤ 31,684) [10]; GWAS: Suzuki et al. 2024 (European population, N_eff ≤ 751,755) [20].

Supplementary Table S2: Complete Results of the Machine Learning Threshold Sensitivity Analysis

Supplementary Table S2. Complete results of ML threshold sensitivity analysis (40 combinations)

Top_N	Vote_K	n_hub	n_overlap	n_lost	n_new	Jaccard
5	2	5	5	7	0	0.4167
5	3	4	4	8	0	0.3333
5	4	4	4	8	0	0.3333
5	5	3	3	9	0	0.2500
10	2	7	7	5	0	0.5833
10	3	5	5	7	0	0.4167
10	4	4	4	8	0	0.3333
10	5	3	3	9	0	0.2500
15	2	12	9	3	3	0.6000
15	3	7	7	5	0	0.5833
15	4	4	4	8	0	0.3333
15	5	3	3	9	0	0.2500
20	2	15	11	1	4	0.6875
20	3	9	9	3	0	0.7500
20	4	4	4	8	0	0.3333
20	5	3	3	9	0	0.2500
25	2	19	11	1	8	0.5500
25	3	9	9	3	0	0.7500
25	4	4	4	8	0	0.3333
25	5	3	3	9	0	0.2500
30	2	24	11	1	13	0.4400
30	3	9	9	3	0	0.7500
30	4	4	4	8	0	0.3333
30	5	3	3	9	0	0.2500
35	2	28	11	1	17	0.3793
35	3	9	9	3	0	0.7500
35	4	4	4	8	0	0.3333
35	5	3	3	9	0	0.2500
40	2	33	11	1	22	0.3235
40	3	10	10	2	0	0.8333
40	4	4	4	8	0	0.3333
40	5	3	3	9	0	0.2500
45	2	37	11	1	26	0.2895
45	3	11	11	1	0	0.9167
45	4	4	4	8	0	0.3333
45	5	3	3	9	0	0.2500
50	2	39	12	0	27	0.3077
50	3	12	12	0	0	1.0000
50	4	4	4	8	0	0.3333
50	5	3	3	9	0	0.2500

Note: Top_N is the cutoff value for RF, XGBoost, and SVM-RFE; Vote_K is the voting threshold (selected by ≥ K algorithms). n_hub: the number of hub genes under that combination; n_overlap: the number in the intersection with the original 12 genes; n_lost: the number lost from the original 12 genes; n_new: the number of newly added genes not among the original 12. Jaccard = |intersection| / |union|. Bold rows indicate the parameters used in the main text (Top-50, Vote ≥ 3, Jaccard = 1.0) and the parameters that first reached Jaccard ≥ 0.75 (Top-20, Vote ≥ 3). LASSO (3 features) and Boruta (4 features) produce fixed outputs. SVM-RFE outputs at most 15 features, so its results no longer increase when Top-N > 15.

Supplementary Table S3: Detailed Instrumental Variable Information for MYOF and RUNX2

Supplementary Table S3. Detailed instrumental variable information for MYOF and RUNX2

Gene	SNP	Chr	Position	EA	OA	Z_eQTL	N_eQTL	β_eQTL	SE_eQTL	P_eQTL	F	β_GWAS	SE_GWAS	P_GWAS	Wald OR	95%CI
MYOF	rs11187158	10	94502429	C	T	5.56	24,071	0.0358	0.00645	2.67×10^{-8}	30.9	-0.0256	0.0060	1.73×10^{-5}	0.489	0.310–0.772
MYOF	rs11594445	10	95083039	T	C	-34.23	25,483	-0.2144	0.00626	1.01×10^{-256}	1172.0	-0.0022	0.0075	0.772	1.010	0.944–1.081
RUNX2	rs3799973	6	44823727	A	G	18.59	31,682	0.1045	0.00562	3.54×10^{-77}	346.0	-0.0152	0.0037	3.98×10^{-5}	0.865	0.752–0.995
RUNX2	rs1200428	6	45518202	T	G	-57.46	31,684	-0.3228	0.00562	3.27×10^{-310}	3302.7	0.0022	0.0046	0.641	0.993	0.960–1.028

IVW pooled results:

Gene	N_IV	Method	β_IVW	SE_IVW	P_IVW	OR_IVW	95%CI	FDR
MYOF	2	IVW	—	—	2.79×10^{-5}	0.999	0.999–1.000	1.68×10^{-4}
RUNX2	2	IVW	—	—	4.20×10^{-5}	0.998	0.998–0.999	1.68×10^{-4}

Note: EA: effect allele (the allele that increases gene expression in the eQTL); OA: other allele. β_eQTL and SE_eQTL were derived from the eQTLGen Zscore and NrSamples: $\beta = Z/\sqrt{N}$, $SE = 1/\sqrt{N}$. F statistic = Z^2. β_GWAS/SE_GWAS: Suzuki 2024 T2D GWAS (EUR, N_eff ≤ 751,755), the log-OR and standard error after alignment of the effect allele. Wald OR = exp(β_GWAS/β_eQTL); 95%CI = exp(Wald ± 1.96×SE_GWAS/|β_eQTL|). The Wald ratios of the two MYOF IVs were in inconsistent directions (rs11187158 OR < 1, protective; rs11594445 OR > 1, no effect), but after IVW pooling P = 2.79×10^{-5} and FDR = 1.68×10^{-4}, with the overall estimate pointing to a protective effect. The two RUNX2 IVs were likewise not fully consistent in direction. This inconsistency reflects the difference in effect size between a single weak IV (rs11187158, F = 30.9) and a strong IV (rs11594445, F = 1172); after the IVW method pools by variance weighting, the result is dominated by the strong IV.

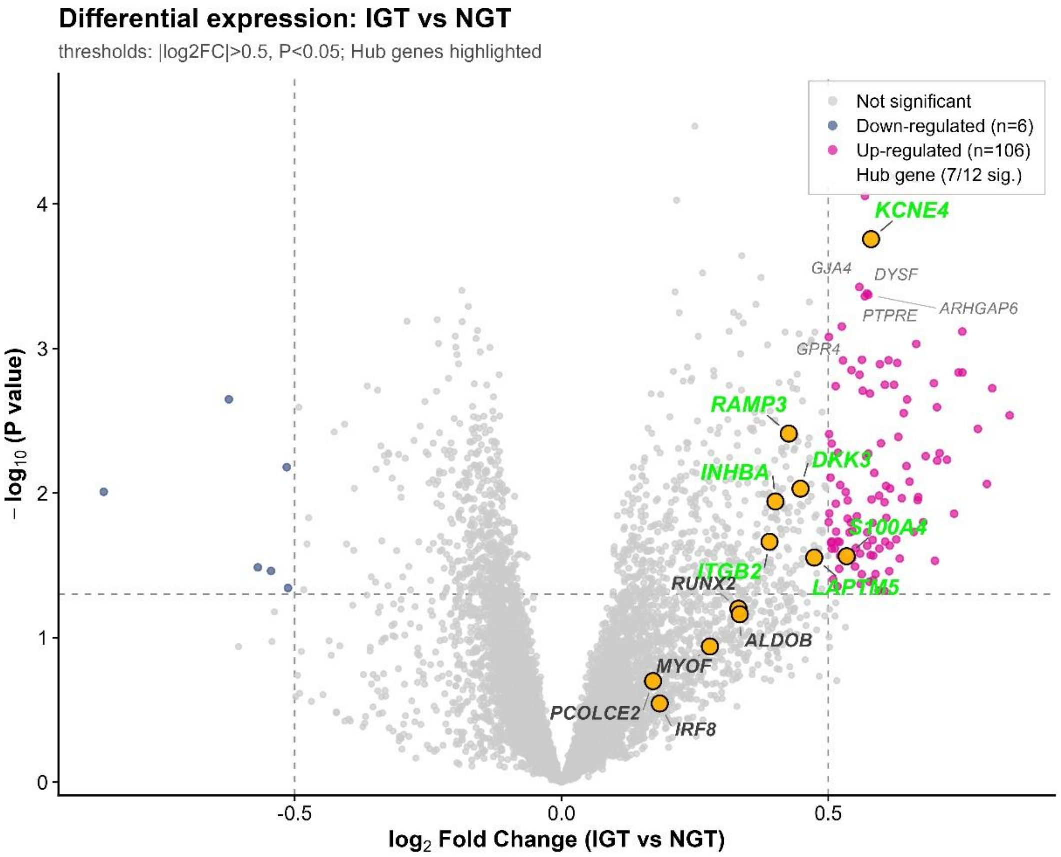

Supplementary Figure S1: Volcano plot of IGT versus NGT differential expression (no significant DEGs)

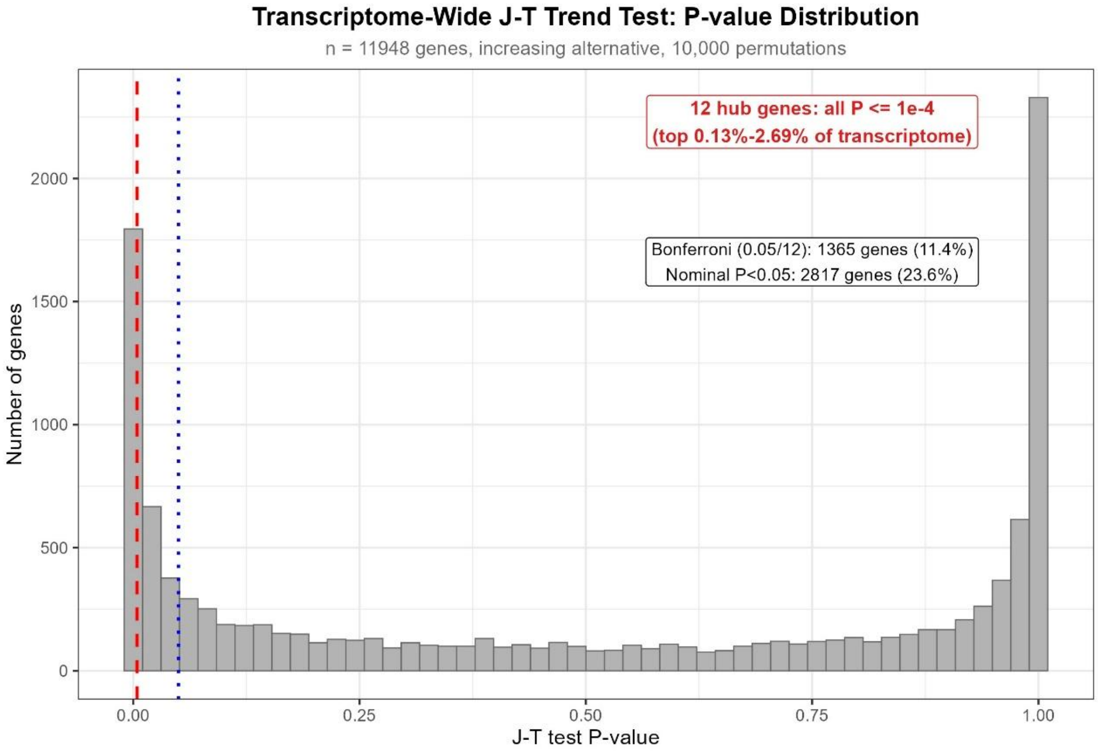

Supplementary Figure S2. Transcriptome-wide Jonckheere–Terpstra trend test P-value distribution histogram (11,948 genes)

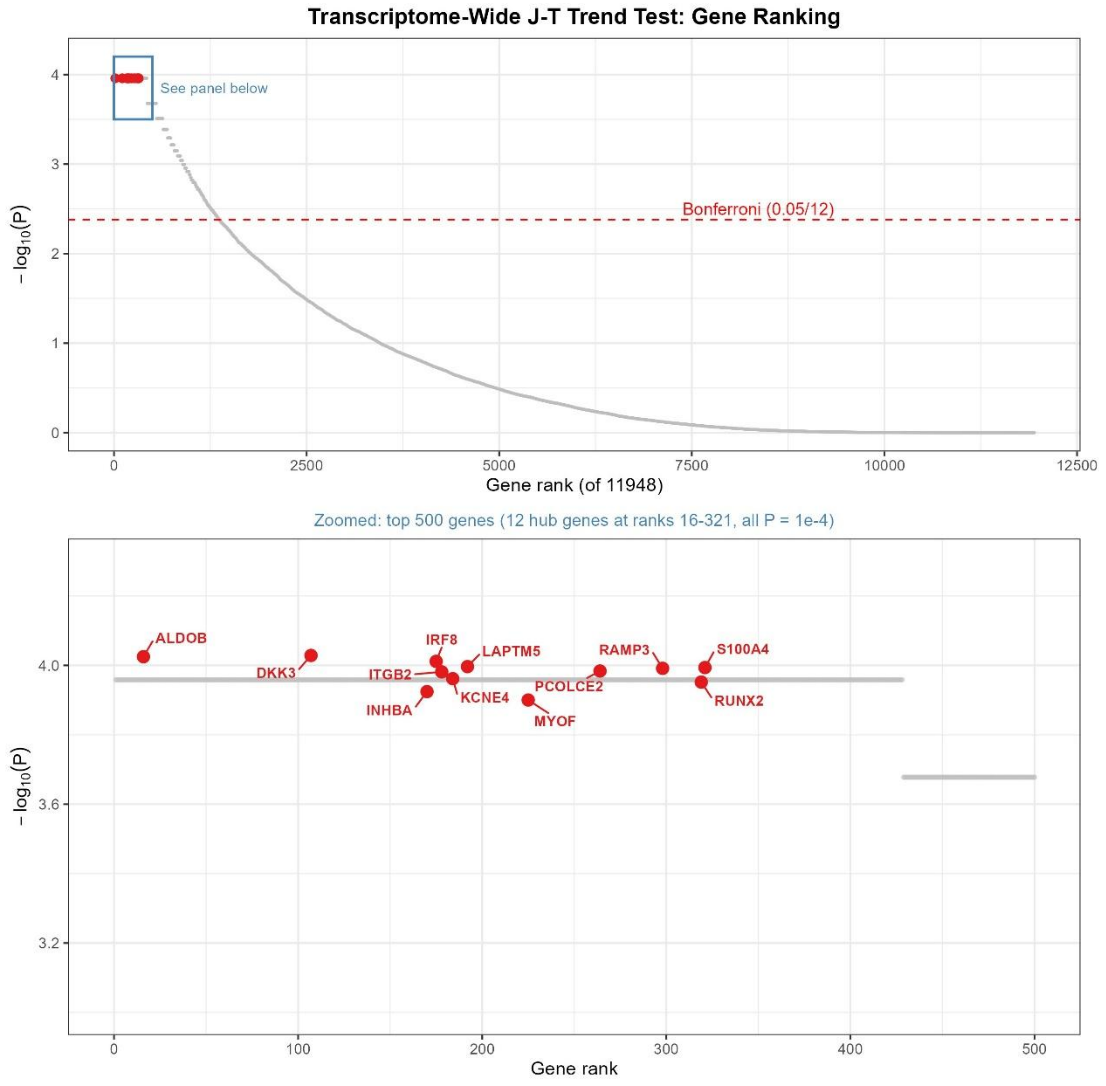

Supplementary Figure S3. Transcriptome-wide J–T test gene ranking plot with the 12 hub genes highlighted
